# A Live-Cell Imaging System for the Identification of Pro- and Anti-Aggregatory α-Synuclein Modulators

**DOI:** 10.1007/s12035-026-06248-2

**Published:** 2026-10-06

**Authors:** Tzukit Tal, Yael Almog, Ortal Iancu Cohen, Lihi Radomir, Dana Bar-On, Ianai Fishbein, Eran Meshorer

**Affiliations:** 1 Department of Genetics, The Institute of Life Sciences, The Edmond J. Safra Campus, The Hebrew University, 9190401 Jerusalem, Israel; 2 Non-Clinical Development Department, Teva Pharmaceutical Industries Ltd, 4250483 Netanya, Israel; 3 The Edmond and Lily Safra Center for Brain Sciences (ELSC), The Hebrew University, The Edmond J. Safra Campus, 9190401 Jerusalem, Israel

**Keywords:** Alpha-synuclein, Aggregation, Bimolecular fluorescence complementation, BiFC, Live-cell imaging, High-content imaging, U373 cells, Synucleinopathies, Drug screening

## Abstract

α-Synuclein (αSyn) aggregation is a defining pathological feature of synucleinopathies, including Parkinson’s disease, dementia with Lewy bodies, and multiple system atrophy (MSA). However, existing in vitro methods to monitor αSyn aggregation often rely on endpoint assays that cannot distinguish monomeric from oligomeric species or capture early aggregation dynamics. Here, we implement an αSyn bimolecular fluorescence complementation (αSyn-BiFC) strategy in a stable human glial-derived cell system optimized for real-time, high-content analysis of early αSyn self-association events and their modulation. In this strategy, αSyn is fused to complementary fragments of the Venus fluorescent protein, enabling real-time visualization of αSyn-αSyn interactions during oligomer formation. Using U373 human astrocytoma cells, a scalable glial-derived cell line previously used in studies of MSA, we established both transient and stable αSyn-BiFC expression systems. We demonstrate that reconstituted Venus fluorescence correlates with pSer129 αSyn immunostaining, supporting the association of the BiFC signal with pathology-related αSyn assemblies. The model responded robustly to multiple stressors known to promote αSyn aggregation, including proteostasis disruption (leupeptin, MG132), ER stress (tunicamycin), and environmental toxins (rotenone), while iron, particularly Fe^3^⁺, also enhanced αSyn self-association. Furthermore, testing two small molecules previously shown to attenuate αSyn aggregation and pathology in vivo revealed a reduction in Venus fluorescence, consistent with reduced αSyn self-association and supporting the platform’s utility for drug discovery screens. By providing a dynamic, sensitive, and physiologically relevant readout of early αSyn assemblies, the αSyn-BiFC model overcomes limitations of conventional assays and offers a scalable framework for mechanistic studies, compound screening, and integration into more disease-relevant neuronal and aging-enhanced models for future synucleinopathies research.

**Supplementary Information:**

The online version contains supplementary material available at https://doi.org/10.1007/s12035-026-06248-2.

## Introduction

Alpha-synuclein (αSyn) is a soluble, intrinsically disordered protein abundantly expressed in the brain [1, 2]. It is the primary aggregated protein in synucleinopathies, including Parkinson’s disease (PD), dementia with Lewy bodies (DLB), and multiple system atrophy (MSA) [3, 4]. In PD and DLB, αSyn aggregates predominantly within neurons to form Lewy bodies, whereas MSA is characterized by the accumulation of αSyn in oligodendrocytes [1]. Misfolded αSyn forms toxic oligomers and fibrils that can propagate between cells in a prion-like manner [5–7]. Accumulating evidence suggests that soluble oligomeric species, rather than mature fibrils, are key mediators of αSyn-induced toxicity [8, 9], although substantial structural and functional heterogeneity exists among oligomer species [10, 11].

To better understand these processes in a controlled and mechanistic manner, experimental systems that recapitulate defined stages of αSyn aggregation and toxicity at the cellular level are required. Accordingly, cell-based in vitro models of synucleinopathies have become essential tools for investigating the molecular and cellular pathways underlying αSyn aggregation and for evaluating potential therapeutic interventions. Early and widely used models rely on intracellular overexpression of wild-type or mutant αSyn (e.g., A53T, A30P, or E46K) [12, 13]. More recent cell-based systems have incorporated inducible expression paradigms and endogenously tagged αSyn reporters, enabling tighter control over αSyn levels and more quantitative analysis of aggregation dynamics [7, 13, 14]. In parallel, complementary models have been developed in which preformed αSyn fibrils (PFFs) are applied to cultured cells to seed templated aggregation and prion-like propagation, allowing controlled initiation of αSyn pathology [15–17]. Collectively, these strategies have been implemented across a range of experimental platforms, including immortalized cell lines such as HEK293, SH-SY5Y and H4 cells [15, 18], primary rodent neurons [7, 16], and human-induced pluripotent stem cell (hiPSC)-derived neurons [19, 20], facilitating detailed interrogation of aggregation kinetics and downstream cellular responses, as reviewed by Delenclos et al. [13].

Modeling MSA is particularly challenging because its pathology is centered on glial, rather than neuronal, mechanisms and because it lacks a known genetic cause. While several in vitro models have been developed to study MSA-related αSyn pathology [21], human-based systems remain limited. Notably, αSyn overexpression in the U373 human astrocytoma cell line has been reported to induce fibrillar αSyn aggregation and cellular stress responses, including increased vulnerability to oxidative insults [21, 22]. While U373 cells do not fully recapitulate oligodendrocyte biology or MSA pathology, these observations support their use as a scalable human glial-derived platform for studying αSyn assembly and pathology-associated cellular responses.

Measuring αSyn aggregation in these models typically relies on endpoint assays such as western blotting, immunocytochemistry of phosphorylated serine 129 (pSer129), or thioflavin T fluorescence. pSer129 is widely used as a marker of pathological aggregation [23] because it is highly enriched in αSyn inclusions in PD, DLB, and MSA. However, it is an imperfect indicator: not all aggregates are phosphorylated at this site, and pSer129 can also occur in soluble, non-aggregated αSyn. These traditional approaches are indirect, lack temporal resolution, and provide limited insight into early aggregation events.

To address these limitations, live-cell imaging strategies have been developed to monitor αSyn assembly and aggregation dynamics in real time. Early live-cell studies relied on αSyn fused to full-length fluorescent proteins such as GFP or YFP to visualize intracellular accumulation, inclusion formation, and toxicity in yeast and mammalian systems [24]. While these reporters provided important insights into αSyn aggregation dynamics and cellular consequences of αSyn overexpression, they do not intrinsically or unambiguously discriminate monomeric non pathological αSyn from toxic small, soluble oligomeric assemblies without additional validation [24]. Within this expanding class of live-cell approaches, several biosensor-based imaging strategies have emerged to selectively report αSyn-αSyn proximity events associated with early oligomer formation [25]. FRET-based αSyn biosensors provide sensitive detection of early conformational changes [26, 27], but they require specialized fluorescence-lifetime instrumentation, careful donor-acceptor calibration, and can suffer from nonspecific background signal [26, 27]. The optogenetic OASIS approach enables precise, light-controlled induction of αSyn aggregation [28], but depends on artificial activation and specialized equipment, which may limit physiological relevance.

Another live-cell strategy is bimolecular fluorescence complementation (BiFC) [29], in which αSyn is fused to complementary fragments of a fluorescent protein that reconstitute fluorescence upon intermolecular interaction [30–33]. When applied to αSyn, BiFC enables visualization of αSyn-αSyn proximity events associated with early oligomer formation and has been validated in cultured cells and in vivo [30–32], where BiFC signal correlates with αSyn-mediated toxicity and neurodegenerative phenotypes. In this context, BiFC provides a relatively straightforward and accessible readout, with low background that enables dynamic monitoring of early αSyn oligomerization without the need for complex instrumentation. BiFC-based αSyn reporters typically produce diffuse cytosolic fluorescence consistent with small and intermediate oligomeric assemblies [32] rather than mature fibrillar inclusions, distinguishing them from conventional aggregation readouts. However, only a subset of αSyn molecules participates in fluorescence complementation, and BiFC signal intensity does not scale linearly with aggregate load, necessitating careful interpretation and complementary validation strategies [33].

Despite their broad use, existing αSyn-BiFC implementations have largely relied on transient expression systems or stable in vivo models optimized for detailed mechanistic investigations rather than quantitative high-throughput screenings. Thus, although αSyn-BiFC is a conceptually established intracellular reporter of αSyn molecular assembly and oligomerization, a gap remains in the availability of stable, human cell–based BiFC platforms optimized for high-throughput, reproducible, real-time monitoring of αSyn self-association and assembly under controlled perturbations and for scalable compound-screening applications.

In this study, we adapt and implement an established αSyn-BiFC strategy into a stable, human glial-derived cellular platform optimized for live-cell, high-content imaging. The αSyn-BiFC approach used here should be viewed as part of a broader progression of intracellular aggregation reporters, evolving from early αSyn-GFP/YFP systems through interaction- and conformation-based probes designed to improve specificity for early misfolded assemblies. Using U373 astrocytoma cells, previously used for studies of αSyn pathology [21, 22], we generated both transient and stable αSyn-BiFC expression systems that enable reproducible, real-time quantification of early αSyn assemblies across large cell populations. This configuration allows systematic interrogation of stressors that promote αSyn assembly and evaluation of candidate anti-aggregatory compounds within a scalable screening-compatible format. Using this system, we examined how various stressors, including tunicamycin, rotenone, and iron, affect αSyn assembly. We also incorporated two clinically investigated small molecules: emrusolmin (Anle138b/TEV 56286), which inhibits αSyn oligomer formation by binding fibrillar and misfolded intermediates [34–38] and minzasolmin (UCB0599; (R)-NPT200-11), which blocks membrane-associated αSyn misfolding at early aggregation steps [39, 40] (nonetheless, Minzasolmin failed its phase 2 clinical trial). Application of these compounds demonstrated the suitability of the BiFC platform for evaluating candidate therapeutics.

By enabling selective detection of early αSyn assemblies, this model overcomes a major limitation of conventional immunostaining, which cannot distinguish monomeric from aggregated states. It provides a dynamic and quantifiable readout suitable for studying cellular and molecular factors that influence αSyn aggregation kinetics. Furthermore, when combined with high-content imaging microscopy, this system offers a powerful platform for screening candidate compounds that may inhibit or modulate αSyn self-association and assembly, with promising implications for therapeutic development in synucleinopathies.

## Materials and Methods

### Statistical Analysis

All statistical analyses were performed in GraphPad Prism. Comparisons between two independent groups were carried out using unpaired *t* tests with Welch’s correction. For comparisons involving three or more independent groups at a single endpoint, data were analyzed using either Welch’s one-way ANOVA with Dunnett’s T3 multiple comparison test (parametric, unequal variances) or, when normality assumptions were not met, Kruskal-Wallis tests followed by Dunn’s multiple comparison test (nonparametric alternative to one-way ANOVA).

For repeated-measures time-course experiments, full trajectories were analyzed using either repeated-measures one-way ANOVA with the Geisser–Greenhouse correction (parametric) or, when data were not normally distributed, the Friedman test with Dunn’s multiple comparison test (nonparametric repeated-measures ANOVA). When endpoint values at 72 h were analyzed separately, these were evaluated using Welch’s one-way ANOVA with Dunnett’s T3 test, or unpaired *t* tests with Welch’s correction for two-group comparisons. Dose-response experiments were analyzed using nonlinear regression (four-parameter variable-slope logistic models) to obtain EC₅₀ values. All statistical tests were two-tailed, and *p* < 0.05 was considered statistically significant. Data are presented as mean ± SEM unless otherwise indicated.

### Plasmid Constructs

We fused the alpha-synuclein (αSyn) coding sequence at its C-terminus to either the Venus N-terminal or C-terminal fragments. We ordered both Venus fragments as gBlocks from Integrated DNA Technologies (IDT), amplified them by PCR, and assembled them into an N174-based viral vector (phDLX1-N174, Addgene #60859, digested with EcoRI and NotI) using the NEBuilder HiFi DNA Assembly Cloning Kit (NEB-E5520S). We replaced the Geneticin resistance cassette in the N174-aSyn-NC vector with a puromycin resistance cassette before viral production.

αSyn-Venus N-terminus:

- atggatgtattcatgaaaggactttcaaaggccaaggagggagttgtggctgctgctgagaaaaccaaacagggtgtggcagaagcagcaggaaagacaaaagagggtgttctctatgtaggctccaaaaccaaggagggagtggtgcatggtgtggcaacagtggctgagaagaccaaagagcaagtgacaaatgttggaggagcagtggtgacgggtgtgacagcagtagcccagaagacagtggagggagcagggagcattgcagcagccactggctttgtcaaaaaggaccagttgggcaagaatgaagaaggagccccacaggaaggaattctggaagatatgcctgtggatcctgacaatgaggcttatgaaatgccttctgaggaagggtatcaagactacgaacctgaagccctcgagGTCGACCTCGACatggtgagcaagggcgaggagctgttcaccggggtggtgcccatcctggtcgagctggacggcgacgtaaacggccacaagttcagcgtgtccggcgagggcgagggcgatgccacctacggcaagctgaccctgaagctgatctgcaccaccggcaagctgcccgtgccctggcccaccctcgtgaccaccctgggctacggcctgcagtgcttcgcccgctaccccgaccacatgaagcagcacgacttcttcaagtccgccatgcccgaaggctacgtccaggagcgcaccatcttcttcaaggacgacggcaactacaagacccgcgccgaggtgaagttcgagggcgacaccctggtgaaccgcatcgagctgaagggcatcgacttcaaggaggacggcaacatcctggggcacaagctggagtacaactacaacagccacaacgtctatatcaccgcctaatctagagggcccgtttaaacccgctgatcagcctcga

αSyn coding seq, VDLD linker, Venus N-terminus, stop codon is underlined

αSyn-Venus C-terminus:

- atggatgtattcatgaaaggactttcaaaggccaaggagggagttgtggctgctgctgagaaaaccaaacagggtgtggcagaagcagcaggaaagacaaaagagggtgttctctatgtaggctccaaaaccaaggagggagtggtgcatggtgtggcaacagtggctgagaagaccaaagagcaagtgacaaatgttggaggagcagtggtgacgggtgtgacagcagtagcccagaagacagtggagggagcagggagcattgcagcagccactggctttgtcaaaaaggaccagttgggcaagaatgaagaaggagccccacaggaaggaattctggaagatatgcctgtggatcctgacaatgaggcttatgaaatgccttctgaggaagggtatcaagactacgaacctgaagccctcgagGTCGACCTCGACaagaacggcatcaaggccaacttcaagatccgccacaacatcgaggacggcggcgtgcagctcgccgaccactaccagcagaacacccccatcggcgacggccccgtgctgctgcccgacaaccactacctgagctaccagtccgccctgagcaaagaccccaacgagaagcgcgatcacatggtcctgctggagttcgtgaccgccgccgggatcactctcggcatggacgagctgtacaagtaatctagagggcccgtttaaacccgctgatcagcctcga

αSyn coding seq, VDLD linker, Venus C-terminus; stop codon is underlined

PCR primers for fragment amplification:

Forward: 5′ caggtgtcgtgaggatccgcatggatgtattcatgaaaggac 3′

Reverse: 5′ gcctcccctacccggtagaatttcgaggctgatcagcgggtttaaacg 3′

### Transient Transfection

We transfected cells using the Lipofectamine 3000 kit (Invitrogen, Cat. #L3000014) according to the manufacturer’s instructions.

### Viral Production and Induction

The ELSC Vector Core Facility at the Hebrew University of Jerusalem produced the viral particles. For viral induction, we mixed cells with the virus and TransduceIT transduction reagent (Mirus), incubated the mixture at 37 °C for 20 min, and centrifuged the cells at 900 × g for 30 min at 30 °C. After centrifugation, we replaced the medium with fresh growth medium and allowed the cells to recover for at least 48 h. After recovery, we maintained the cells in medium supplemented with geneticin (150 µg/ml) and puromycin (0.5 µg/ml) to ensure selection for both constructs.

### Cell Culture

We cultured U373 cells in EMEM (ATCC 30–2003) supplemented with 10% FBS and 1% penicillin–streptomycin at 37 °C and 5% CO₂. We changed the medium twice per week and passaged cells once per week using trypsin.

### Live Imaging

We performed live-cell imaging using a Molecular Devices high-content imaging instrument. Images were acquired every 3 h using the FITC channel, capturing nine sites per well in black µClear 96-well plates (Corning, Cat. #655090). Most experiments were imaged for 72 h. The emrusolmin experiment (Fig. 6C) and the corresponding cell-count analysis (Supplementary Fig. 4D) were extended to 144 h to evaluate sustained effects of compound treatment on the αSyn-BiFC signal.

### Live Imaging Analysis

Image analysis was carried out in ImageXpress (Molecular Devices) using the Multi-Wavelength Cell Scoring module. Venus intensity was quantified for each segmented cell, and two fixed thresholds were applied:

- a low threshold that identifies all cells with detectable FITC-range fluorescence, including some background autofluorescence;
- a high threshold that defines the strongly expressing population corresponding to αSyn-BiFC complementation.

The percentage of Venus-positive cells was calculated as:

$$\%\;\mathrm{Venus}\;\mathrm{positive}\;\mathrm{cells}=\:(\mathrm{number}\;\mathrm{of}\;\mathrm{high}\;\mathrm{threshold}\;\mathrm{cells}/\mathrm{total}\;\mathrm{number}\;\mathrm{of}\;\mathrm{Venus}\;\mathrm{expressing}\;\mathrm{cells})\times\:100.$$

 

The same thresholds were applied consistently across all wells and time points within each experiment. To assess the robustness of the analysis, representative datasets were re-analyzed using multiple fluorescence thresholds spanning a broad intensity range. Although absolute percentages of Venus-positive cells varied according to threshold selection, the relative responses to treatment remained consistent (Supplementary Fig. 1), indicating that the biological conclusions were not dependent on a specific threshold value. In all graphs shown in the “Results” section where Y-axis label is “% Venus positive cells,” it refers specifically to the fraction of high-expressing cells, normalized to the total number of Venus-expressing cells in each well. This metric reflects the proportion of cells undergoing BiFC complementation rather than absolute fluorescence intensity.

### Theoretical Estimation of BiFC Interfaces Within αSyn Oligomers

To interpret the αSyn-BiFC signal, we modeled the expected number of productive Venus-complementing interaction interfaces within an oligomer. In this BiFC configuration, fluorescence occurs only when αSyn molecules carrying complementary Venus fragments (VN and VC) form a direct interaction interface. Assuming random mixing of constructs and a linear oligomer topology, an oligomer of size *n* contains (*n* – 1) αSyn-αSyn interfaces. The expected number of VN–VC interfaces is therefore:

$$E\left[\#VNVC\right]=2{p}_{VN}{p}_{VC}\left(n-1\right),$$

where ${p}_{VN}$ and ${p}_{VC}$ denote the intracellular fractions of VN- and VC-tagged αSyn, respectively. The factor $2{p}_{VN}{p}_{VC}$ represents the probability that a given interface is heterotypic (either VN–VC or VC–VN), which is required for Venus complementation. Consequently, the expected BiFC intensity per oligomer scales as:

$$I\left(n\right)\propto 2{p}_{VN}{p}_{VC}\left(n-1\right).$$

Within the physiologically relevant size range of αSyn oligomers (approximately 8–30 monomers), this relationship behaves approximately linearly, supporting the interpretation that the BiFC signal primarily reflects early and intermediate oligomeric assemblies.

### Immunostaining

We fixed cells with 4% formaldehyde and 4% sucrose in physiological buffer for 15 min at room temperature with gentle shaking. After two DPBS washes, we blocked and permeabilized the cells for 30 min using 1% BSA, 10% goat serum, 0.3% Triton X-100, and 0.05% sodium azide in DPBS. We diluted primary antibodies in 1:5 blocking buffer and incubated the cells overnight at 4 °C with gentle shaking. After three DPBS washes, we applied secondary antibodies (1:400 dilution) together with Hoechst 33342 (3.3 µg/ml) for 30–60 min in the dark, followed by three additional DPBS washes.

### Soluble and Insoluble Protein Fractionation and Western Blot Analysis

Cells were cultured in 12-well plates and treated as indicated. Following treatment, cells were washed twice with ice-cold PBS and harvested for soluble/insoluble fractionation. Cells were lysed in Triton X-100-containing lysis buffer (50 mM Tris-HCl, pH 7.5, 150 mM NaCl, 1 mM EDTA, 1% Triton X-100 supplemented with protease and phosphatase inhibitors) and incubated on ice for 30 min. Lysates were centrifuged at 20,000 × g for 30 min at 4 °C. The resulting supernatant was collected as the soluble protein fraction. Pellets were washed once with ice-cold TBS and resuspended in SDS-containing lysis buffer (50 mM Tris-HCl, pH 7.5, 2% SDS supplemented with protease and phosphatase inhibitors). Samples were sonicated for 1 min in a bath sonicator, heated at 95 °C for 5 min, and centrifuged at 20,000 × g for 10 min. The resulting supernatant was collected as the insoluble protein fraction. Equal volumes of soluble and insoluble fractions were separated by SDS-PAGE and transferred to PVDF membranes. To improve retention of αSyn species, membranes were fixed after transfer in PBS containing 4% paraformaldehyde and 0.01% glutaraldehyde for 30 min, washed three times with TBS-T, and subsequently blocked. Membranes were probed with antibodies against total αSyn and phosphorylated αSyn (pSer129) as indicated. GAPDH was used as a loading control for soluble fractions, and total protein staining was used for normalization of insoluble fractions.

### Compound Treatments

We treated cells with the indicated compounds by diluting each stock solution directly into growth medium immediately before use. We kept the final DMSO concentration at ≤ 0.1% for all conditions, including controls. For time-course experiments, we added compounds at the start of imaging and maintained continuous exposure for 72 h. For dose-response experiments, we treated cells for 72 h. For iron experiments, we freshly prepared FeSO₄ and FAC in DDW and added them to cells immediately after preparation to minimize oxidation.

### Flow Cytometry

We analyzed Venus fluorescence using a Cytek® Aurora 4-Laser spectral flow cytometer. Venus was excited with the 488 nm blue laser, and its signal was quantified from the unmixed fluorescence assigned to the B2 channel, which corresponds to the expected Venus emission range. Spectral unmixing was performed in SpectroFlo™ using single-stained Venus-positive controls and unstained cells to generate reference spectra. Cells were gated by forward and side scatter to remove debris and doublets, and Venus-positive events were defined relative to the unstained baseline. For each sample, 10,000–20,000 live singlet events were acquired. Data were analyzed in FlowJo to quantify mean and median Venus intensity and to visualize full fluorescence distributions, allowing assessment of heterogeneity in Venus expression or aggregation state.

### Reagents

Detailed tables of cell culture reagents, compounds, and immunostaining antibodies are included below.

Cell Culture Reagents

**Table:** 

Reagent	Provider	Catalog number
EMEM	ATCC	30–2003
DMEM/F-12	Sigma-Aldrich	6421
FBS	Gibco	A5256701
Pen Strep	Gibco	15140-122
Trypsin	Gibco	25300-062
Geneticin	Gibco	10131-035
Puromycin	InvivoGen	ant-pr-1
DPBS, no calcium, no magnesium	Thermo Fisher Scientific	14190-094

Compounds

**Table:** 

Reagent	Provider	Catalog number	Concentration/diluant
Leupeptin Ready Made Solution	Sigma-Aldrich	SAE0153	5 mg/ml in non-freezing solution
MG-132	Sigma-Aldrich	M7449	10 mM in DMSO
Tunicamycin	Sigma-Aldrich	SML1287	5 mg/ml in DMSO
Paraquat dichloride hydrate	Supelco	36541	100 mM in DDW
Dieldrin	Sigma-Aldrich	33491	100 mM in DMSO
Rotenone	Supelco	45656	2 mM in DMSO
Iron(II) sulfate heptahydrate	Sigma-Aldrich	F8633	10 mM in DDW
Ammonium iron(III) citrate	Sigma-Aldrich	F5879	10 mM in DDW

Immunostaining Antibodies and Reagents

**Table:** 

Reagent	Provider	Catalog number	Dilution
Anti-Alpha-synuclein (phospho S129) antibody [EP1536Y]	Abcam	Ab51253	1:500
Purified Mouse Anti-α-Synuclein	BD	610787	1:1000
Goat anti-Rabbit IgG (H + L) Cross-Adsorbed Secondary Antibody, Alexa Fluor™ 568	Thermo Fisher Scientific	A11011	1:400
Goat anti-Mouse IgG (H + L) Cross-Adsorbed Secondary Antibody, Alexa Fluor™ 568	Thermo Fisher Scientific	A11004	1:400
Goat anti-Mouse IgG (H + L) Highly Cross-Adsorbed Secondary Antibody, Alexa Fluor™ 647	Thermo Fisher Scientific	A21236	1:400
Hoechst 33342	Thermo Fisher Scientific	H3570	1:3000
Formaldehyde solution about 37%	Sigma-Aldrich	1.04003	4%
D-(+)-Sucrose	Sigma-Aldrich	S0389	4%
BSA	Sigma-Aldrich	A7906	1%
Donor goat serum	Biological Industries	04–009-1	10%
Triton	Sigma-Aldrich	X-100	0.3%

## Results

### The αSyn-BiFC System Reports Pathology-Associated αSyn Assembly

**Fig. 1:**
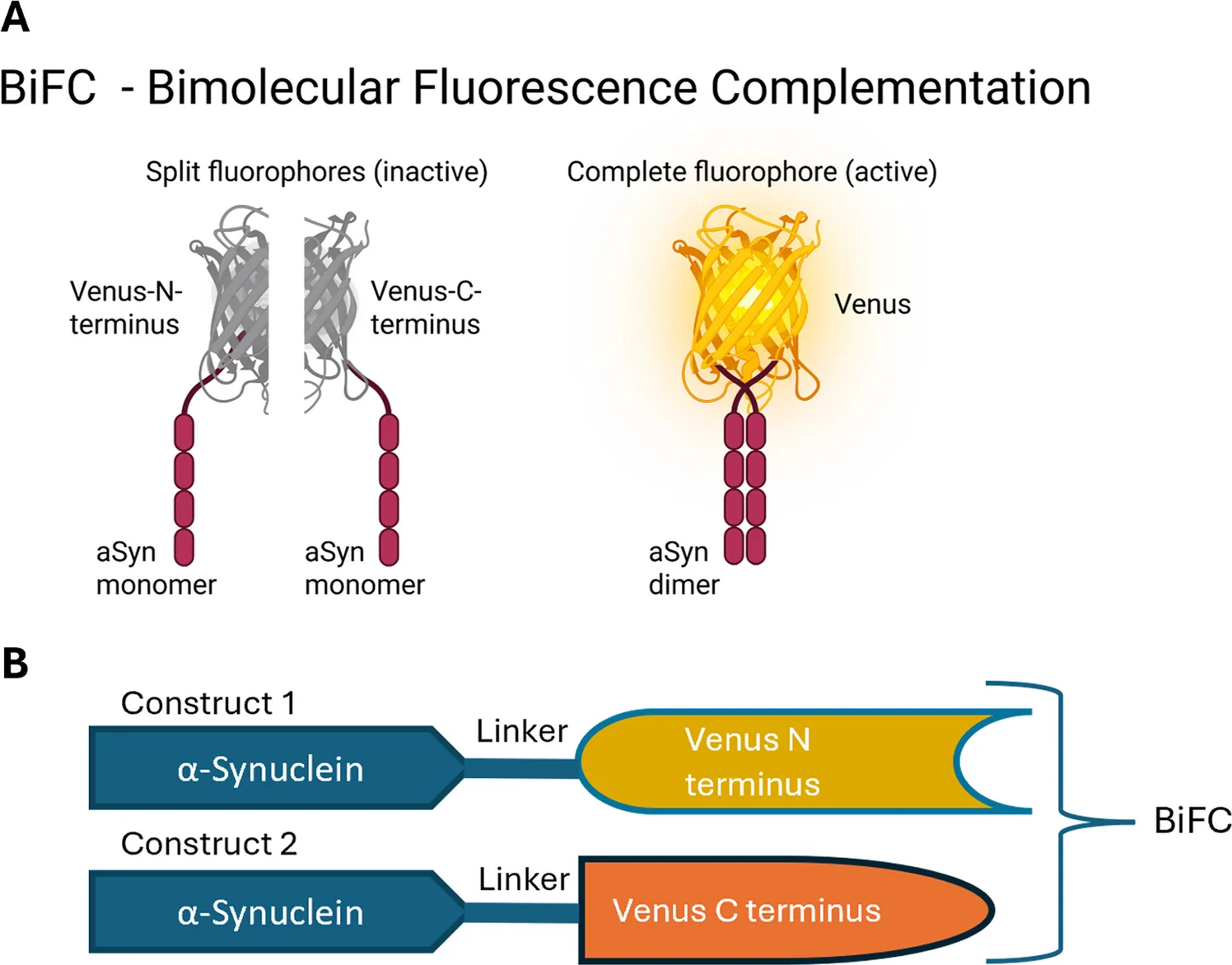
The αSyn-BiFC method. **A** Schematic representation of the αSyn-BiFC assay at the protein level, illustrating Venus fragment complementation upon αSyn-αSyn interaction or oligomerization. Created with BioRender.com. **B** Design of the expression constructs encoding αSyn fused to either the N-terminal or C-terminal fragment of Venus, enabling reconstitution of fluorescence upon αSyn dimerization or aggregation

BiFC enables detection of protein–protein proximity through the reconstitution of a split fluorescent protein when its two fragments are brought together. In the context of αSyn, reconstitution of Venus fluorescence is expected to occur primarily when αSyn molecules interact or form early oligomeric assemblies, whereas monomeric αSyn should produce little or no signal. Based on this principle, we used the system to visualize potential αSyn-αSyn oligomerization in living cells (Fig. 1A). Importantly, the BiFC signal reflects the number of αSyn interaction interfaces formed within oligomers rather than the absolute mass of aggregated material. Because complementation occurs only when the two Venus fragments meet, the intensity approximately increases with oligomer size within the small-to-intermediate oligomeric regime observed for αSyn in cells. Thus, increases in Venus signal in response to aggregation-promoting conditions are interpreted as enhanced formation of early and intermediate αSyn oligomers, rather than as a direct measurement of mature fibrils.

To establish the αSyn-BiFC system, we designed two plasmid vectors to enable transient co-expression of αSyn fused to complementary halves of the Venus fluorescent protein (αSyn-VN and αSyn-VC), separated by a short flexible linker (Fig. 1B). To test the system, we first transiently co-expressed these two constructs into U373 cells. As control, we used transient expression of αSyn fused to GFP.

**Fig. 2:**
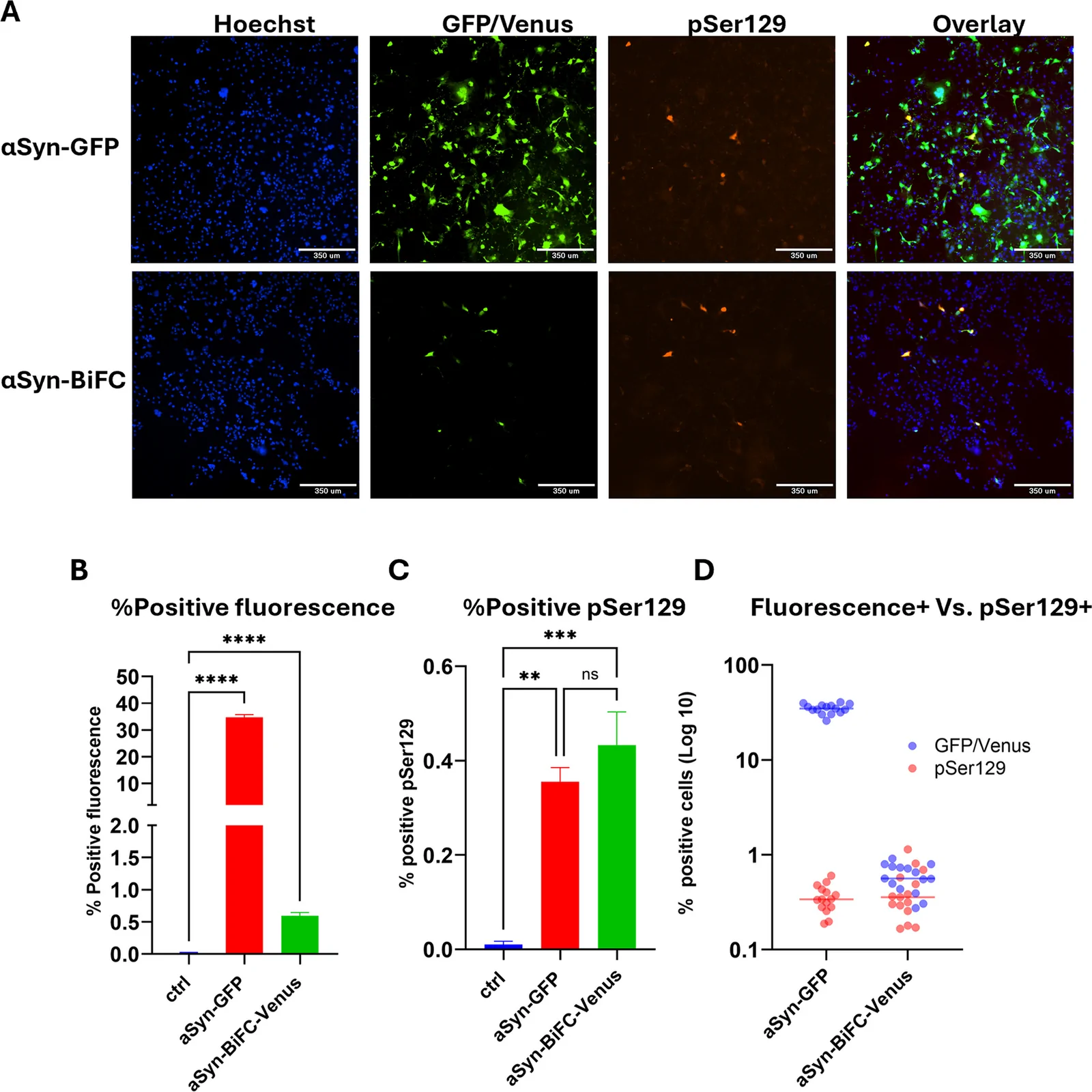
The αSyn-BiFC system accurately reflects αSyn pathology.** A** Representative images of cells transfected with αSyn-GFP or αSyn-BiFC constructs. Following fixation, cells were immunostained for phosphorylated αSyn (pSer129, red) and counterstained with Hoechst (blue). GFP or reconstituted Venus fluorescence is visible without additional staining (green). **B** Percentage of GFP-positive (αSyn-GFP, red) or Venus-positive (αSyn-BiFC-Venus, green) cells versus control (blue) (Welch’s one-way ANOVA with Dunnett’s T3 multiple-comparisons, αSyn-GFP *p* < 0.001; αSyn-BiFC *p* < 0.001). Data represents mean ± SEM (*n* = 15 per group). **C** pSer129-positive cell percentage in αSyn-GFP (red) or αSyn-BiFC-Venus (green) relative to control (blue) (Kruskal–Wallis test with Dunn’s multiple comparisons, αSyn-GFP vs. ctrl *p* < 0.01; αSyn-BiFC vs. ctrl *p* < 0.001; αSyn-GFP vs. αSyn-BiFC ns). Data represents mean ± SEM (*n* = 15 per group). **D** Distribution of reporter-positive (blue) and pSer129-positive (red) cells on a log scale, illustrating relative abundance and spread. Each group consisted of 15 wells (96-well plate), with 16 imaging sites per well. Imaging was performed using a high-content imaging system (Molecular Devices) and analyzed using MetaXpress (Multi-Wavelength Cell Scoring)

Forty-eight hours post-transfection, we fixed and immunostained the cells for phosphorylated serine 129 (pSer129). As expected, fluorescence microscopy revealed markedly higher GFP signal in αSyn-GFP-transfected cells compared to Venus fluorescence in αSyn-BiFC-transfected cells (Fig. 2A, B). This difference demonstrates that Venus fluorescence requires proximity-driven reconstitution of its two halves, reflecting αSyn-αSyn interactions, whereas GFP fluorescence is present in all αSyn-GFP molecules regardless of aggregation state. The detection of Venus implies that spontaneous αSyn-αSyn interactions occur at a low frequency under basal conditions, likely driven by αSyn overexpression even in the absence of an external aggregation trigger. Despite the lower overall Venus signal, pSer129 staining was observed at comparable levels in both αSyn-GFP and αSyn-BiFC conditions (Fig. 2A, C, and D).

Notably, while pSer129-positive cells constituted only a fraction of all αSyn-GFP positive cells, there was almost complete overlap between Venus-positive cells and pSer129-positive cells (Fig. 2A and D). This co-localization indicates that Venus fluorescence in the BiFC system is associated with pathology-related αSyn assemblies, confirming that BiFC‑positive cells reliably represent the population undergoing pathological αSyn phosphorylation.

### Generation and Characterization of a Stable αSyn-BiFC Expressing Cell Line

To enable long-term studies without repeated transfection, we next generated a stable U373 cell line expressing our αSyn-BiFC system. We packaged the constructs in two lentiviral vectors, each encoding one half of the αSyn-BiFC construct and transduced our cells. As control, we also transduced each lentiviral vector separately (αSyn-VN and αSyn-VC). Following antibiotic selection, we obtained stable populations expressing either both constructs or each construct alone.

**Fig. 3:**
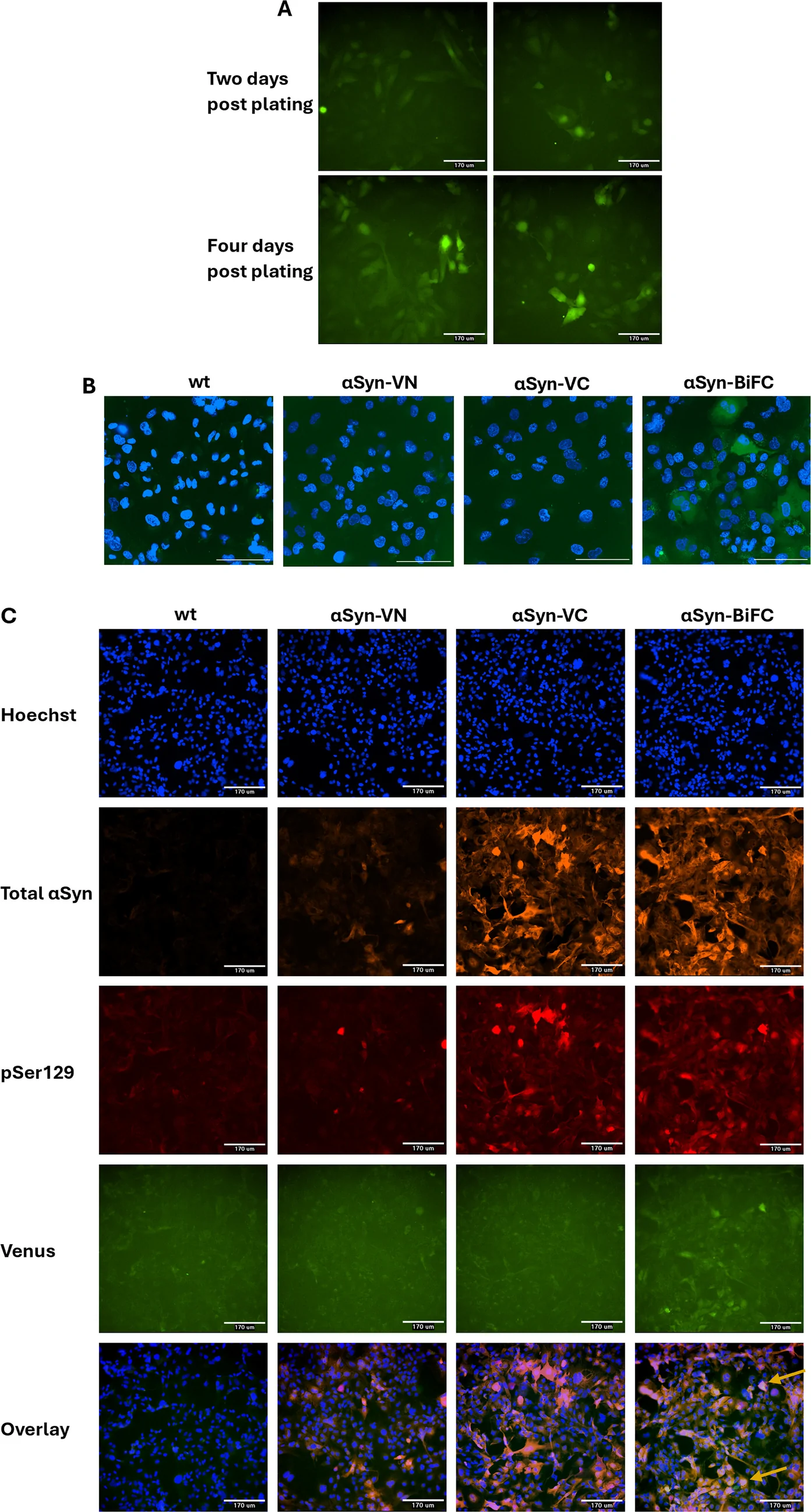

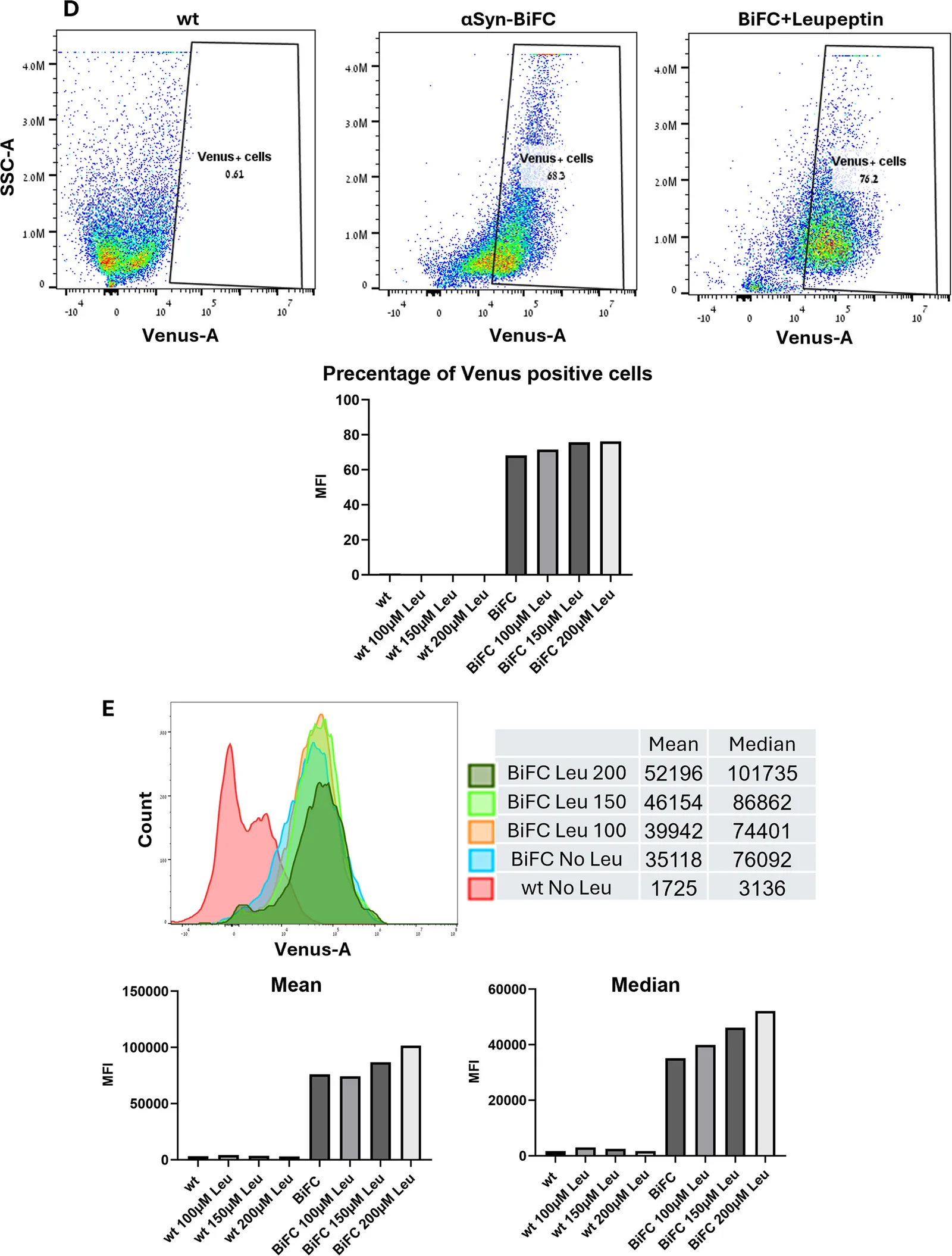
Venus fluorescence reports early aggregation and αSyn pathology in stable αSyn-BiFC U373 cells. **A** Two representative live-cell images of U373 cells expressing the αSyn-BiFC constructs, showing reconstituted Venus fluorescence 2 (top) and 4 (bottom) days after plating (FITC channel). Images were acquired using a high-content imaging system (Molecular Devices). **B** Representative images of live cells stained with Hoechst to visualize nuclei and imaged for Venus fluorescence. Constructs include αSyn-VN (αSyn fused to the N-terminal Venus fragment) and αSyn-VC (αSyn fused to the C-terminal Venus fragment). Scale bar = 100 µm. Images were acquired using a confocal microscopy (BioTek Cytation C10 by Agilent). **C** Representative images of U373 wild type (wt), αSyn-BiFC, αSyn VN (αSyn fused to the N-terminal Venus fragment), and αSyn VC (αSyn fused to the C-terminal Venus fragment) cells. Total αSyn (orange), pSer129 αSyn (red), and Venus αSyn (green) are shown. Orange arrows indicate regions where Venus signal overlaps with pSer129 and total αSyn. Images were acquired using a high-content imaging system (Molecular Devices). **D** Flow-cytometry analysis showing Venus-positive αSyn-BiFC cells. Side scatter versus Venus intensity demonstrates a broad fluorescence range. **E** Histogram analysis indicates that treatment with leupeptin increases both the mean and median Venus fluorescence intensity, consistent with enhanced intracellular accumulation of αSyn-BiFC signal

Fluorescence microscopy revealed detectable Venus signal in the αSyn-BiFC stable cell line, as early as 6 h after plating. By 96 h post-plating, most cells exhibited low basal levels of Venus fluorescence, while a subset displayed higher fluorescence intensity even in the absence of an external trigger (Fig. 3A and B). This suggests, as in the previous transient expression experiment, that spontaneous αSyn interactions occur at a low frequency under basal conditions. As expected for a complementation-dependent system, no Venus fluorescence was detected in cells expressing only αSyn-VN or αSyn-VC, even though both total αSyn and pSer129 were readily detected (Fig. 3B and C). Venus fluorescence was generally diffuse and evenly distributed throughout the cytoplasm of positive cells, without conspicuous subcellular localization. Immunostaining for total αSyn demonstrated widespread cellular expression in all cell lines, including those lacking detectable Venus signal and controls expressing only αSyn-VN or αSyn-VC (Fig. 3C). While total αSyn levels in αSyn-VC cells were comparable to those in αSyn-BiFC cells, expression levels in αSyn-VN cells were lower.

Consistent with the immunostaining results, Western blot analysis confirmed lower expression of the αSyn-VN construct relative to αSyn-VC and αSyn-BiFC cells (Supplementary Fig. 2). Western blot analysis further demonstrated expression of the expected full-length αSyn-Venus fusion proteins without evidence of major degradation products (Supplementary Fig.  2 A). Immunostaining for pSer129 further showed that many Venus-positive cells were also positive for pSer129, particularly those exhibiting higher Venus intensity, although not all Venus-positive cells displayed detectable pSer129 staining (Fig. 3C). Western blot analysis further confirmed the presence of pSer129-positive αSyn in αSyn-BiFC, αSyn-VN, and αSyn-VC cells (Supplementary Fig. 2B). Consistently, 96 h post-plating, flow cytometry analysis revealed a broad distribution of Venus fluorescence intensities, suggesting the presence of multiple αSyn assembly states within the population (Fig. 3D and E). These results indicate that Venus fluorescence correlates with pathological αSyn in a substantial fraction of cells, while also capturing earlier or less advanced aggregation states.

### Validation of the αSyn-BiFC System Using Potential Aggregation-Inducing Compounds

**Fig. 4:**
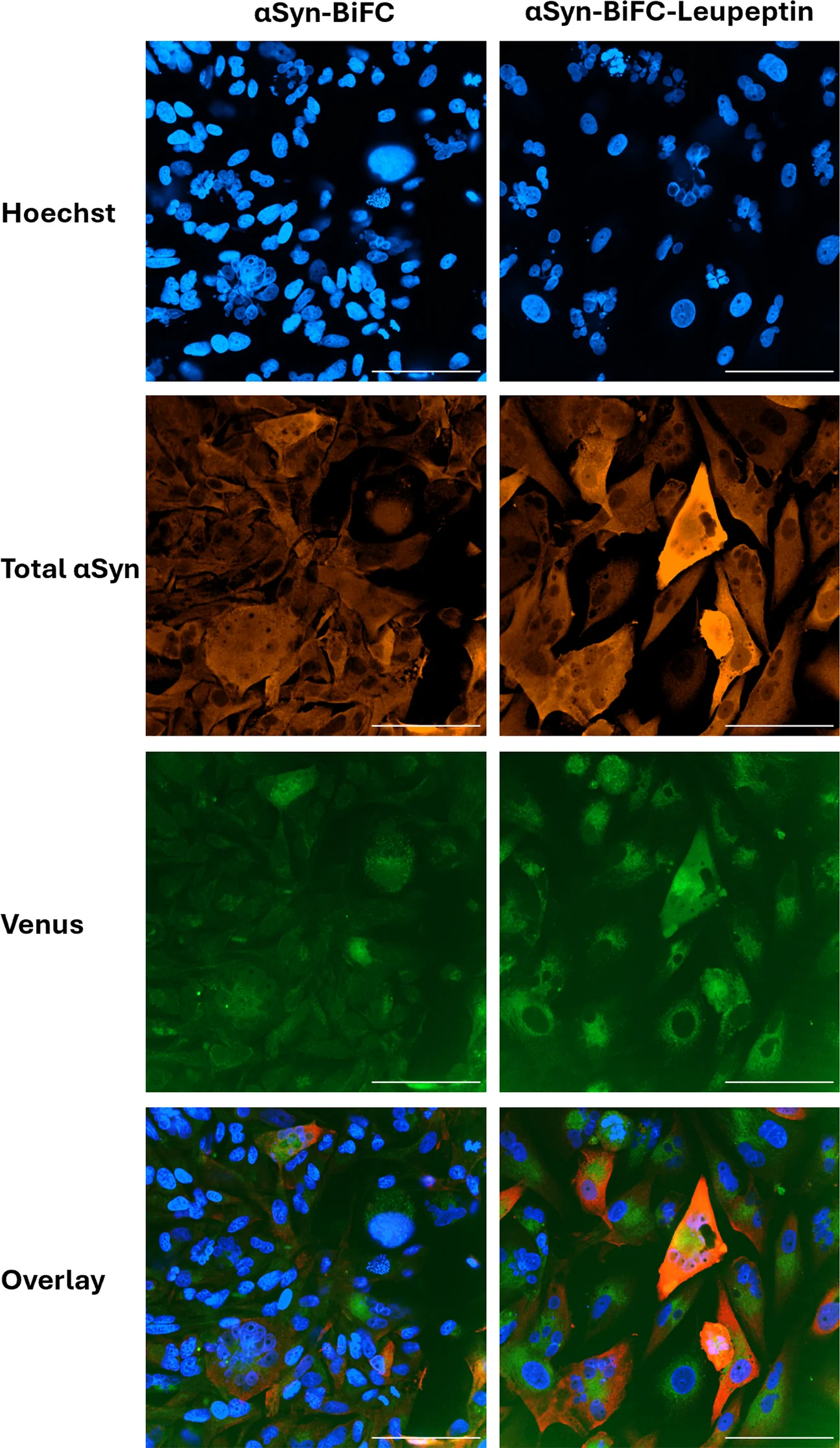
Leupeptin treatment increases αSyn-BiFC signal. Representative images of U373 αSyn-BiFC and αSyn-BiFC cells following leupeptin treatment. Total αSyn (orange) and Venus αSyn (green). Scale bar = 100 µm. Images were acquired using a confocal microscopy (BioTek Cytation C10 by Agilent)

**Fig. 5:**
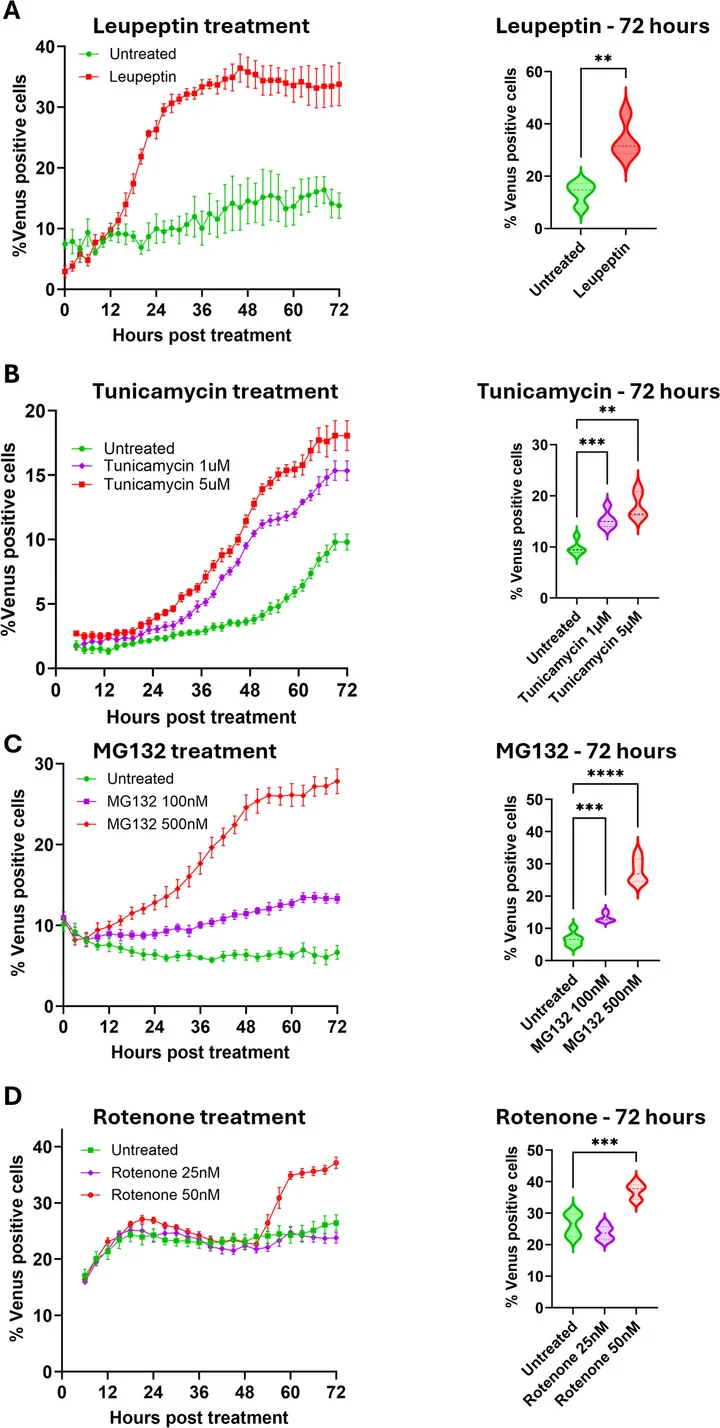
Validation of the αSyn-BiFC system using aggregation-inducing compounds. **A** Real-time Venus fluorescence of untreated (green) or Leupeptin-treated (200 µM, red) U373 αSyn-BiFC cells over 72 h (Wilcoxon matched pairs signed rank test, two tailed; *p* < 0.001). Right: Violin plot of Venus fluorescence at 72 h (unpaired *t* test with Welch’s correction; *p* < 0.005). Data represents mean ± SEM (*n* = 4 per group). **B** Real-time Venus fluorescence of untreated (green) or tunicamycin-treated (1 µM, purple; 5 µM, red) U373 αSyn-BiFC cells over 72 h (Friedman test with Dunn’s multiple comparisons, *p* < 0.001 at 1 µM; *p* < 0.001 at 5 µM). Right: Violin plot of Venus fluorescence at 72 h (Welch’s one-way ANOVA with Dunnett’s T3 multiple comparisons, *p* < 0.001 at 1 µM; *p* < 0.005 at 5 µM). Data represents mean ± SEM (*n* = 5 per group). **C** Real-time Venus fluorescence of untreated (green) or MG132-treated (100 nM, purple; 500 nM, red) U373 αSyn-BiFC cells over 72 h (Friedman test with Dunn’s multiple comparison test vs. untreated; *p* < 0.005 at 100 nM; *p* < 0.001 at 500 nM). Right: Violin plot of Venus fluorescence at 72 h (Welch’s one way ANOVA with Dunnett’s T3 multiple comparisons, *p* < 0.001 at 100 nM; *p* < 0.001 at 500 nM). Data represents mean ± SEM (*n* = 6 per group). **D** Real-time Venus fluorescence of untreated (green) or rotenone-treated (25 nM, purple; 50 nM, red) U373 αSyn-BiFC cells over 72 h (Friedman test with Dunn’s multiple comparisons, ns at 25 nM; *p* < 0.05 at 50 nM). Right: Violin plot of Venus fluorescence at 72 h (Welch’s one way ANOVA with Dunnett’s T3 multiple comparisons, ns at 25 nM; *p* < 0.001 at 50 nM). Data represents mean ± SEM (*n* = 6 per group)

To assess whether the αSyn-BiFC system responds to physiologically relevant triggers of αSyn aggregation, we treated the stable U373 αSyn-BiFC cells with a panel of compounds selected to model key cellular stress pathways implicated in synucleinopathies pathogenesis. These included compounds impairing protein degradation pathways (leupeptin [41], MG132 [42, 43]), an inducer of ER stress (tunicamycin [44, 45]), and rotenone [46, 47], a mitochondrial complex I inhibitor that is also widely used as a pesticide and associated with environmental risk factors for PD. Several compounds induced a clear increase in both the percentage of Venus-positive cells and Venus fluorescence intensity (Figs. 3D and E, 4, and 5), indicating increased αSyn-BiFC complementation and αSyn assembly. Notably, compounds that interfere with protein degradation pathways were particularly effective. In this live-cell high-content imaging assay, only Venus fluorescence was recorded.

To quantify aggregation, we applied a two-threshold segmentation approach: a low-intensity threshold that captures all detectable cellular objects (which may include minimal autofluorescence) to represent the total cell population and a higher threshold that identifies strongly Venus-positive cells. The ratio between these two populations was expressed as the percentage of cells exhibiting strong αSyn-BiFC signal. Similar treatment-dependent effects were observed across a range of fluorescence thresholds, demonstrating the robustness of the analysis approach (Supplementary Fig. 1). Leupeptin, a broad-spectrum serine/cysteine protease inhibitor [48], robustly increased αSyn-BiFC signal (Fig. 4) over the entire time course (Wilcoxon matched-pairs signed-rank test, two-tailed; *p* < 0.0001), with endpoint values ~ 2.4 fold higher than untreated (Fig. 5A; unpaired *t*-test with Welch’s correction; *p* < 0.005). Consistently, flow cytometry analysis showed that leupeptin elevated mean and median Venus fluorescence intensity 72 h post treatment (Fig. 3D and E).

Tunicamycin, an inducer of ER stress through inhibition of N-linked glycosylation [44], increased αSyn-BiFC signal consistent with enhanced αSyn assembly, by up to ~ twofold at the final time point (Welch’s one-way ANOVA with Dunnett’s T3 multiple-comparisons, *p* < 0.001 at 1 µM; *p* < 0.005 at 5 µM). Across the full-time course, tunicamycin induced a dose-dependent and significant increase at both concentrations (Fig. 5B; Friedman test with Dunn’s multiple comparisons, *p* < 0.001 at 1 µM; *p* < 0.001 at 5 µM). Similarly, MG132 [42], a proteasome inhibitor, significantly increased αSyn-BiFC signal in a dose-dependent manner at both 100 nM and 500 nM concentrations (Fig. 5C; Friedman test with Dunn’s multiple-comparisons, *p* < 0.005 at 100 nM; *p* < 0.001 at 500 nM), consistent with impaired proteasomal clearance of αSyn.

We further tested a panel of environmental toxins, specifically herbicides and pesticides, including dieldrin, rotenone, and paraquat. These compounds have been previously linked to αSyn aggregation in various in vitro and in vivo studies, as well as in patients with synucleinopathies [49]. Paraquat and Dieldrin had minimal effects on αSyn aggregation in this system (data not shown). Rotenone, on the other hand, increased the percentage of Venus-positive cells at 50 nM across the full-time course (Fig. 5D; Friedman test with Dunn’s multiple comparisons, ns at 25 nM; *p* < 0.05 at 50 nM) and at the final time point (Fig. 5D; Welch’s one-way ANOVA with Dunnett’s T3 multiple comparisons, ns at 25 nM; *p* < 0.001 at 50 nM), while lower concentrations had no detectable effect and higher concentrations were cytotoxic, indicating a very narrow window of feasible concentrations in this assay.

To provide an orthogonal assessment of αSyn aggregation, we performed soluble and insoluble fractionation followed by Western blot analysis using antibodies against total αSyn and phosphorylated αSyn (pSer129). Aggregation-promoting conditions increased the accumulation of αSyn in the insoluble fraction (Supplementary Figs. 2, 3B and D) and were accompanied by enhanced pSer129 immunoreactivity (Supplementary Figs. 2B, 4 A and C), consistent with the formation of pathology-associated αSyn assemblies. All aggregation-promoting treatments produced statistically significant increases in insoluble αSyn relative to vehicle controls, although the magnitude of the effect varied between treatments, with leupeptin producing the most pronounced increase (Supplementary Fig. 3B, D). In the soluble fraction, leupeptin significantly increased αSyn levels relative to vehicle controls, whereas MG132, tunicamycin, rotenone, and ferric iron (FAC) did not produce statistically significant changes (Supplementary Fig.  3 A, C).

Consistent with its known role as a lysosomal protease inhibitor, leupeptin increased αSyn levels in both the soluble and insoluble fractions. By impairing αSyn clearance, leupeptin is expected to increase the overall cellular αSyn burden, resulting in the accumulation of both soluble αSyn and insoluble aggregation-associated species. In addition, leupeptin-treated samples exhibited diffuse αSyn-immunoreactive smearing in the insoluble fraction, consistent with the accumulation of heterogeneous higher-molecular-weight αSyn species (Supplementary Figs. 2B and 3B). Together, these findings support the interpretation that increases in Venus fluorescence reflect enhanced αSyn assembly under aggregation-promoting conditions. Western blot analysis further showed that the αSyn-VN and αSyn-VC fusion proteins remained stable following treatment with aggregation-promoting stimuli, with no evidence of treatment-induced degradation or loss of the full-length fusion proteins (Supplementary Fig. 2).

### Evaluation of Therapeutic Compounds in the αSyn-BiFC System

**Fig. 6:**
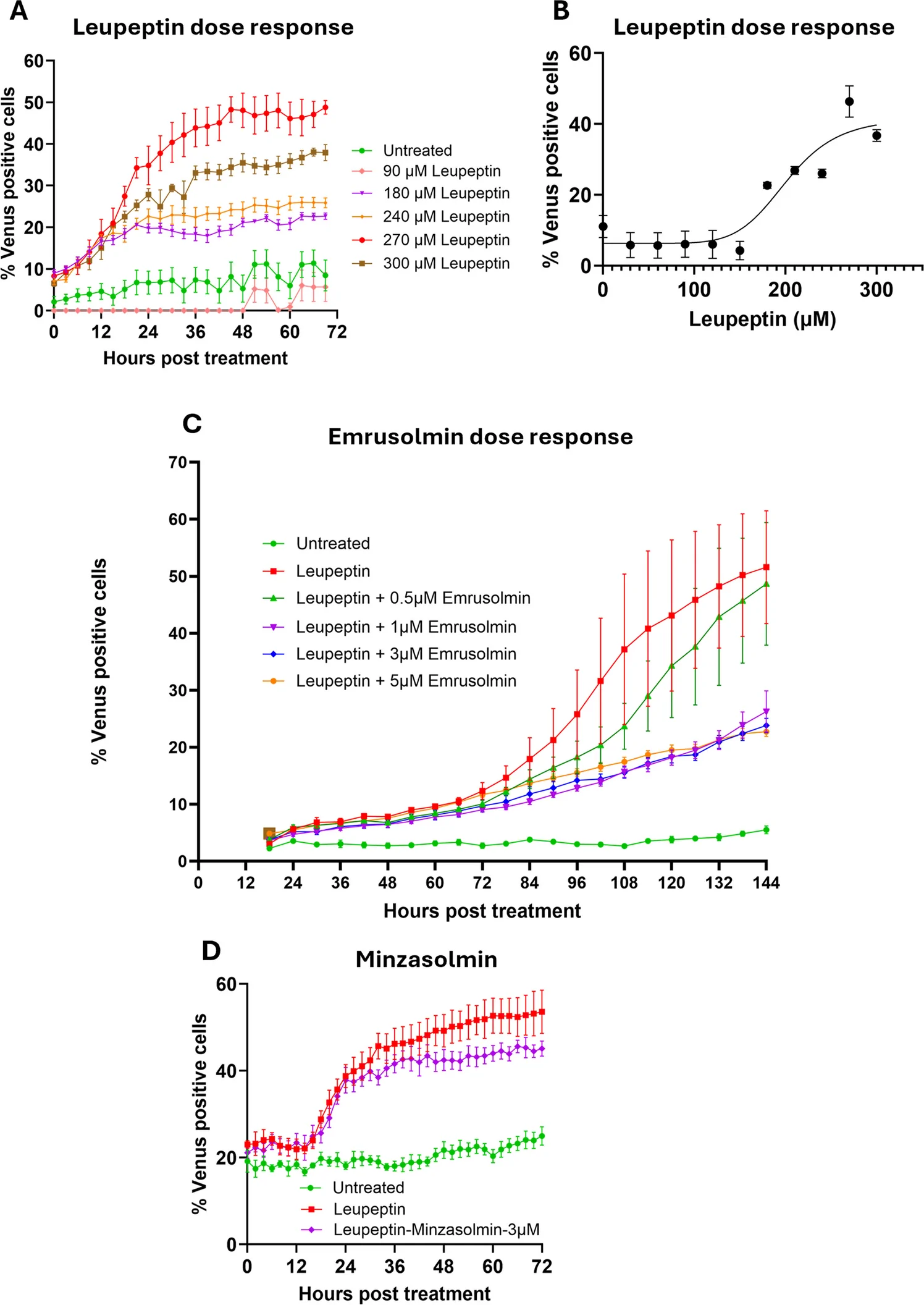
Therapeutic compounds reduce Venus fluorescence in the αSyn-BiFC system. **A** Dose–response of leupeptin treatment (0–300 µM) (Friedman test with Dunn’s multiple comparisons, ns at 90 µM; *p* < 0.05 at 180; *p* < 0.001 at 240 µM; *p* < 0.001 at 270 µM; *p* < 0.001 at 300 µM). Data represents mean ± SEM (*n* = 5 per group). **B** EC₅₀ estimation (0–300 µM). Nonlinear regression variable slope (four-parameter) produced an EC₅₀ of 201.6 µM. Data represents mean ± SEM (*n* = 5 per concentration). **C** Dose response of emrusolmin treatment (0.5–5 µM). U373 αSyn-BiFC cells were pretreated with emrusolmin 2 h before leupeptin challenge (Friedman test with Dunn’s multiple comparisons vs. leupeptin; ns at 0.5 µM; *p* < 0.001 at 1 µM; *p* < 0.001 at 3 µM; *p* < 0.05 at 5 µM). Data represents mean ± SEM (*n* = 6 per group). **D** U373 αSyn-BiFC cells were pretreated with minzasolmin (3 µM) 2 h before leupeptin challenge (Friedman test with Dunn’s multiple comparisons vs. leupeptin; *p* < 0.001). Data represents mean ± SEM (*n* = 5 per group). Imaging was performed using a high-content imaging system (Molecular Devices) and analyzed using MetaXpress (Multi-Wavelength Cell Scoring). The percentage of Venus-positive cells was calculated as the number of high-intensity Venus cells divided by the total Venus-expressing population

To establish a controlled aggregation-inducing condition suitable for testing therapeutic compounds, we first characterized the dose-response of leupeptin. Leupeptin was selected as the challenge stressor because it produced the fastest and most robust induction of αSyn assembly. Although prolonged leupeptin exposure substantially reduced cell number, leupeptin provided a consistent and reproducible increase in the αSyn-BiFC readout, enabling evaluation of potential modulators of αSyn assembly. Leupeptin showed a clear dose-dependent increase, with significant elevations at 180–300 µM (Fig. 6A) and an estimated EC₅₀ of 201.6 µM (Fig. 6B). Although the overall trend was dose-dependent, the response at 300 µM was slightly lower than at 270 µM, likely reflecting the onset of mild cytotoxicity at the highest concentration. We therefore used the EC₅₀ concentration of leupeptin to provide a robust yet sub-maximal aggregation stimulus for evaluating potential therapeutic effects.

Next, we wished to evaluate our system’s ability to detect compounds that reduce αSyn-BiFC signal associated with αSyn assembly under this challenge condition. We examined two small-molecule candidates previously reported to decrease pathological αSyn aggregation and cellular toxicity in vivo [36, 38, 39]. Emrusolmin, a small molecule that inhibits αSyn oligomer formation, was assessed in a dose-response experiment (Fig. 6C). It produced a concentration-dependent reduction in Venus-positive cells, with a modest non-significant decrease at 0.5 µM and progressively stronger reductions at 1–5 µM (Fig. 6C). These effects were statistically significant at 1–5 µM concentrations (Friedman test with Dunn’s multiple comparisons, ns at 0.5 µM; *p* < 0.001 at 1 µM; *p* < 0.001 at 3 µM; *p* < 0.05 at 5 µM). Quantification of total cell number showed that, although leupeptin treatment reduced cell number relative to vehicle-treated cells, co-treatment with emrusolmin did not further reduce cell number compared with leupeptin alone (Supplementary Fig. 4D), indicating that the reduction in Venus-positive cells is unlikely to be explained by additional cell loss. Furthermore, soluble/insoluble fractionation and Western blot analysis demonstrated that emrusolmin slightly reduced the accumulation of insoluble αSyn and pSer129-positive αSyn species under leupeptin challenge conditions (Supplementary Figs. 2 and 4A–C), supporting the interpretation that the decrease in Venus fluorescence reflects reduced αSyn assembly.

We next evaluated Minzasolmin, a small molecule that blocks membrane-associated αSyn misfolding, in the leupeptin-challenge assay (Fig. 6D). Minzasolmin at 3 µM significantly reduced Venus-positive cells compared with leupeptin across the full-time course (Friedman test with Dunn’s multiple-comparison test; *p* < 0.001). Together, these findings demonstrate that the αSyn-BiFC system can detect dose-dependent inhibition of αSyn assembly-associated BiFC signal.

### Iron Enhances αSyn Aggregation in the BiFC System

**Fig. 7:**
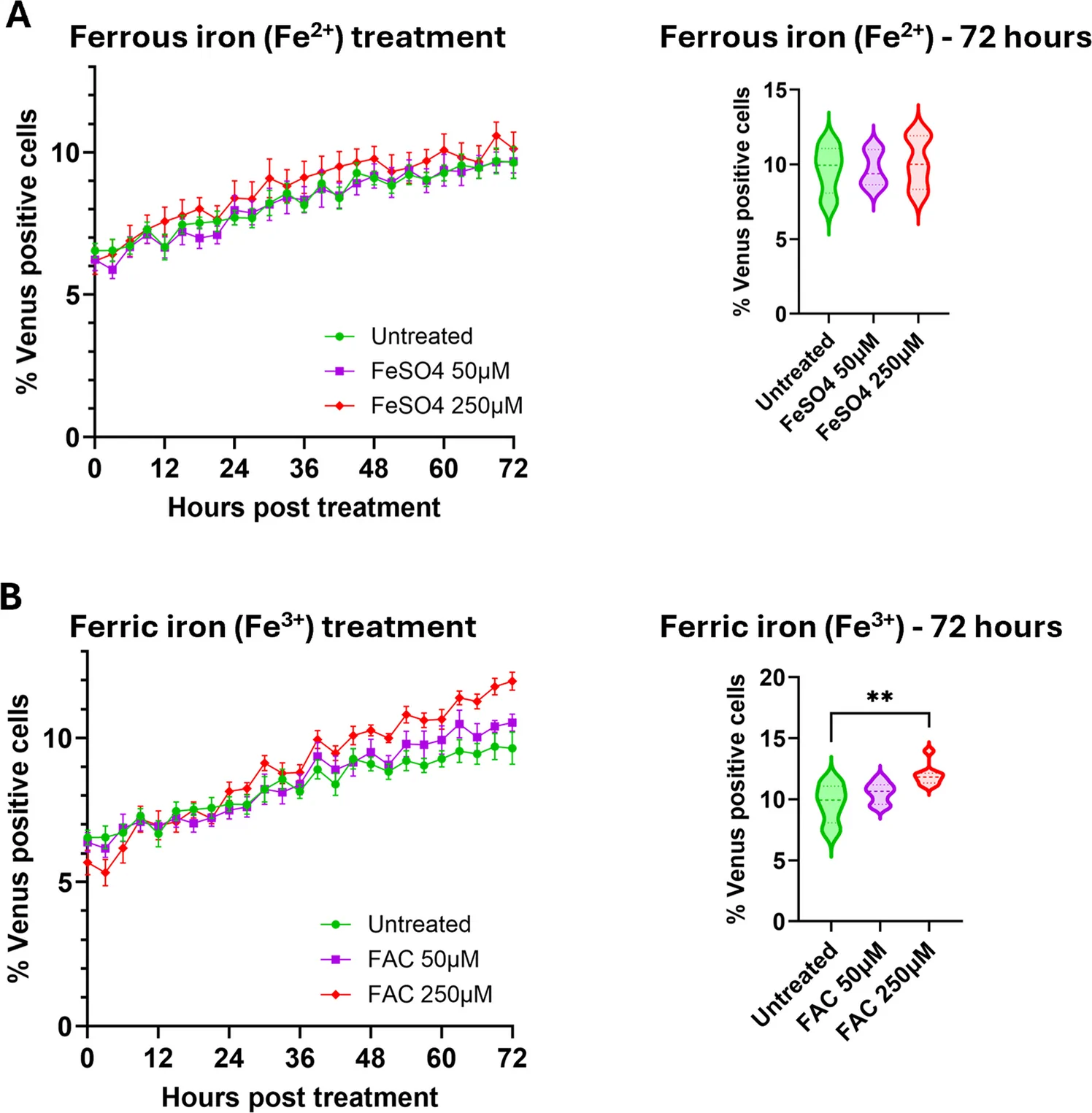
Iron enhances αSyn aggregation in the BiFC system. **A** Real-time Venus fluorescence of untreated (green) or Fe^2^⁺ (FeSO₄)-treated (50 µM, purple; 250 µM, red) U373-αSyn-BiFC cells over 72 h (Friedman test with Dunn’s multiple comparisons, ns at 50 µM; *p* < 0.001 at 250 µM). Right: Violin plot of Venus fluorescence at 72 h (Welch’s one-way ANOVA with Dunnett’s T3 multiple comparison test; ns at 50 µM and 250 µM). Data represents mean ± SEM (*n* = 8 per group). **B** Real-time Venus fluorescence of untreated (green) or Fe^3^⁺ (FAC)-treated (50 µM, purple; 250 µM, red) U373-αSyn-BiFC cells over 72 h (Friedman test with Dunn’s multiple comparisons, ns at 50 µM; *p* < 0.005 at 250 µM). Right: Violin plot of Venus fluorescence at 72 h (Welch’s one-way ANOVA with Dunnett’s T3 multiple comparison test; ns at 50 µM; *p* < 0.005 at 250 µM). Data represents mean ± SEM (*n* = 8 per group). Imaging was performed using a high-content imaging system (Molecular Devices) and analyzed using MetaXpress (Multi-Wavelength Cell Scoring). The percentage of Venus-positive cells was calculated as the number of high-intensity Venus cells divided by the total Venus-expressing population

Iron accumulation is a well-documented pathological feature in multiple system atrophy (MSA) and other synucleinopathies, where it is thought to contribute to αSyn aggregation and neurotoxicity [50, 51]. Both ferrous (Fe^2^⁺) and ferric (Fe^3^⁺) iron species have been implicated in promoting αSyn misfolding, oxidative stress, and fibril formation. To examine iron-mediated effects in the αSyn-BiFC system, we treated the cells with ferrous sulfate (Iron(II) sulfate heptahydrate; FeSO₄; Fe^2^⁺) or ferric ammonium citrate (Ammonium iron(III) citrate; FAC; Fe^3^⁺). FeSO₄ had a modest effect on αSyn aggregation (Fig. 7A; Friedman test with Dunn’s multiple-comparisons, ns at 50 µM; p < 0.001 at 250 µM), likely reflecting rapid oxidation under culture conditions.

FAC produced a modest but reproducible increase in Venus fluorescence at 250 µM across the full-time course (Fig. 7B; Friedman test with Dunn’s multiple-comparisons, ns at 50 µM; *p* < 0.005 at 250 µM), and increased the endpoint readout by ~ 25% vs. untreated (Fig. 7B; Welch’s one-way ANOVA with Dunnett’s T3 multiple-comparisons, ns at 50 µM; *p* < 0.005 at 250 µM).

## Discussion

The αSyn-BiFC approach employed here builds on more than a decade of work establishing BiFC as a robust reporter of early αSyn oligomerization in cells and in vivo [30–33]. Rather than introducing a new aggregation principle, our study focuses on refining this established methodology into a stable, human glial-derived system compatible with long-term live imaging and automated high-content analysis. This distinction is important, as many prior BiFC implementations were designed for mechanistic validation or qualitative imaging, rather than for scalable perturbation or compound-screening workflows. Accordingly, we do not propose αSyn-BiFC as a new conceptual approach for studying αSyn aggregation; instead, our contribution lies in demonstrating that, when implemented as a stable human cell platform, BiFC can serve as a sensitive, reproducible, and scalable readout of early aggregation dynamics suitable for systematic screening applications.

Within this framework, the αSyn-BiFC system described in this study provides a practical and dynamic platform for investigating molecular mechanisms of αSyn assembly and aggregation, a central pathological feature of synucleinopathies including PD, DLB, and MSA. By enabling real-time visualization of αSyn-αSyn interactions in living cells, the system overcomes key limitations of conventional endpoint assays, which cannot distinguish monomeric from assembly-associated αSyn species. The correlation between Venus fluorescence and pSer129 immunostaining supports the specificity of the BiFC signal for pathology-associated αSyn assemblies and validates its use as a reliable readout of early aggregation states.

The establishment of a stable U373 BiFC cell line enables long-term, reproducible studies without repeated transfections. U373 cells offer several advantages: as an immortalized human glioblastoma astrocytoma line originating from the central nervous system, they retain glial features that provide a more physiologically relevant environment than non-brain-derived lines such as HEK293. At the same time, they maintain the practicality, proliferation rate, and reproducibility required for long-term imaging and high-throughput workflows. This combination of physiological relevance and technical robustness provides a strong foundation for a scalable αSyn-BiFC platform. While U373 cells are not bona fide oligodendrocytes, they provide a scalable glial-derived platform and have previously been used in studies of αSyn pathology relevant to MSA [21, 22].

The system proved sensitive to multiple aggregation-promoting conditions, including proteostasis disruption (leupeptin, MG132), ER stress (tunicamycin), and environmental toxins (rotenone), consistent with known contributors to αSyn pathology. The same sensitivity enabled detection of reductions in αSyn aggregation following treatment with emrusolmin and minzasolmin, demonstrating that the assay can capture both pro- and anti-aggregatory effects. The assay’s compatibility with automated high-content imaging further supports its use in medium- to high-throughput screening formats. The stable signal, simple readout, and ability to scale from 96-well to 384-well plates enable efficient evaluation of large compound libraries with minimal optimization.

Prior αSyn-BiFC studies provide important context for our findings. Lázaro et al. [32] reported predominantly diffuse BiFC signals with rare inclusion-like structures, supporting the interpretation that BiFC preferentially reports early oligomeric species rather than mature fibrils. Dominguez-Meijide et al. [52] employed αSyn-BiFC to investigate cell-to-cell transmission using transient co-culture paradigms; however, variability in expression levels and signal intensity limited quantitative and longitudinal analyses in that setting. By contrast, the stable U373 αSyn-BiFC platform described here minimizes expression heterogeneity and enables continuous, automated monitoring across extended time courses, allowing direct and reproducible comparison of aggregation dynamics across experimental conditions and pharmacological perturbations.

Iron treatment, particularly ferric iron, produced a modest but statistically significant increase in the αSyn-BiFC readout. This finding was supported by a corresponding increase in insoluble αSyn levels, suggesting a limited but detectable enhancement of αSyn assembly in this model under the conditions tested. Differences between ferrous and ferric responses likely reflect their distinct redox properties. FeSO₄ (Fe^2+^) produced no detectable effect at 50 µM and a modest increase at 250 µM, consistent with limited stability of ferrous iron under culture conditions. Ferric iron (Fe^3^⁺) induced a reproducible aggregation-enhancing effect, with 250 µM FAC significantly increasing Venus fluorescence across the full 72-h time course and elevating the endpoint readout by ~ 25%. Notably, the FAC time-course curve does not appear to reach a plateau by 72 h, suggesting a progressively accumulating, slow-onset aggregation response. This pattern is consistent with ferric-driven oxidative stress emerging after prolonged exposure, once intracellular buffering capacity becomes saturated [53].

One of the key advantages of this system is its adaptability. The BiFC constructs can be transferred to additional models such as SH-SY5Y cells or iPSC-derived neurons, enabling studies in more disease-relevant cell types.

Aging-related modifications can further enhance the biological relevance of the αSyn-BiFC system. We previously showed that progerin expression in hPSC-derived neurons induces transcriptional aging signatures relevant to neurodegeneration [54]. Similar aging phenotypes can be modeled through telomerase inhibition [55], direct conversion of aged somatic cells to iNs [56], or exposure to oxidative and mitochondrial stress [57]. In addition, αSyn PFFs are widely used to seed endogenous aggregation and propagate pathology in neuronal models [58]. Incorporating the αSyn-BiFC system into iPSC-derived neurons, aging-enhanced models, or PFF-seeded cultures would enable real-time visualization of seeded aggregation under physiologically relevant conditions.

Although BiFC provides a straightforward, sensitive readout of early αSyn interactions, it also has limitations: the fluorescent signal may not scale linearly with aggregate load, only a subset of αSyn participates in complementation [33], and the method lacks structural resolution. In addition, reductions in Venus fluorescence should be interpreted with caution, as some compounds could theoretically affect Venus fluorophore maturation, fluorescence intensity, or complementation of the VN and VC fragments independently of their effects on αSyn assembly. For this reason, confirmation using orthogonal aggregation assays remains important, particularly when evaluating candidate therapeutic compounds. Another limitation is some degree of potential spontaneous Venus complementation, which may contribute to the observed background signal. Since we did not include non-interacting VN/VC fusion controls, this possibility cannot be ruled out. Nevertheless, the treatment-dependent effects observed in the present study were supported by orthogonal validation using soluble/insoluble fractionation and pSer129 detection. In addition, we did not evaluate aggregation-deficient or interaction-disrupting αSyn mutants, which could provide further validation of the specificity of the BiFC signal and strengthen the interpretation that the observed fluorescence changes are driven by αSyn self-association. Incorporation of non-interacting split-Venus controls and aggregation-deficient αSyn mutants represents an important direction for future studies.

These considerations highlight the value of combining BiFC with complementary techniques such as ELISA for aggregated αSyn, Thioflavin T staining, or high-resolution microscopy.

In summary, the αSyn-BiFC U373 system offers a sensitive, specific, and scalable method for studying αSyn aggregation dynamics in living cells. It enables identification of aggregation-promoting stressors, evaluation of environmental and genetic risk factors, and assessment of candidate therapeutics. Its compatibility with automated high-content imaging supports integration into medium- to high-throughput pipelines. Furthermore, the BiFC constructs can be readily implemented in neuronal or glial models, including SH-SY5Y cells and iPSC-derived systems, providing broad opportunities to study αSyn biology in more disease-relevant contexts. Together, these features position the αSyn-BiFC system as a versatile and powerful tool for advancing translational research in synucleinopathies.

## Supplementary Information

Below is the link to the electronic supplementary material.

ESM1 (PDF 2.35 MB)

## Data Availability

No datasets were generated or analysed during the current study
