# Supplementary material for: A Live-Cell Imaging System for the Identification of Pro- and Anti-Aggregatory α-Synuclein Modulators
Source: Mol Neurobiol. 2026 Oct 6;63(1):949. doi: 10.1007/s12035-026-06248-2 (PMC13642413; doi:10.1007/s12035-026-06248-2)
Supplement: Supplementary file 1 [file 12035_2026_6248_MOESM1_ESM.pdf]

Supplementary Fig. 1. Robustness of αSyn-BiFC analysis across fluorescence intensity thresholds

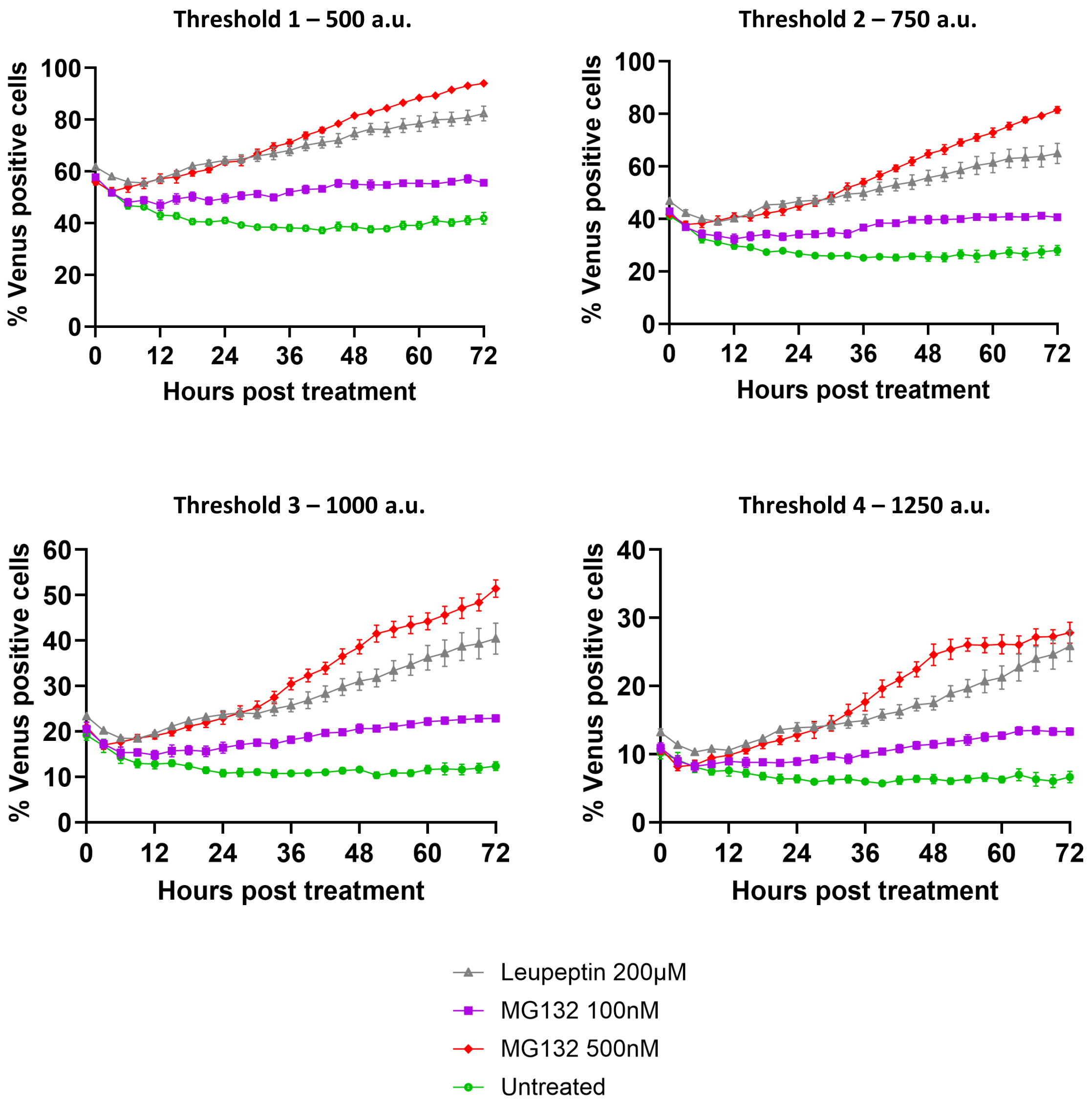

Supplementary Fig. 2. Characterization of αSyn-BiFC, αSyn-VN and αSyn-VC cell lines and stability of fusion proteins

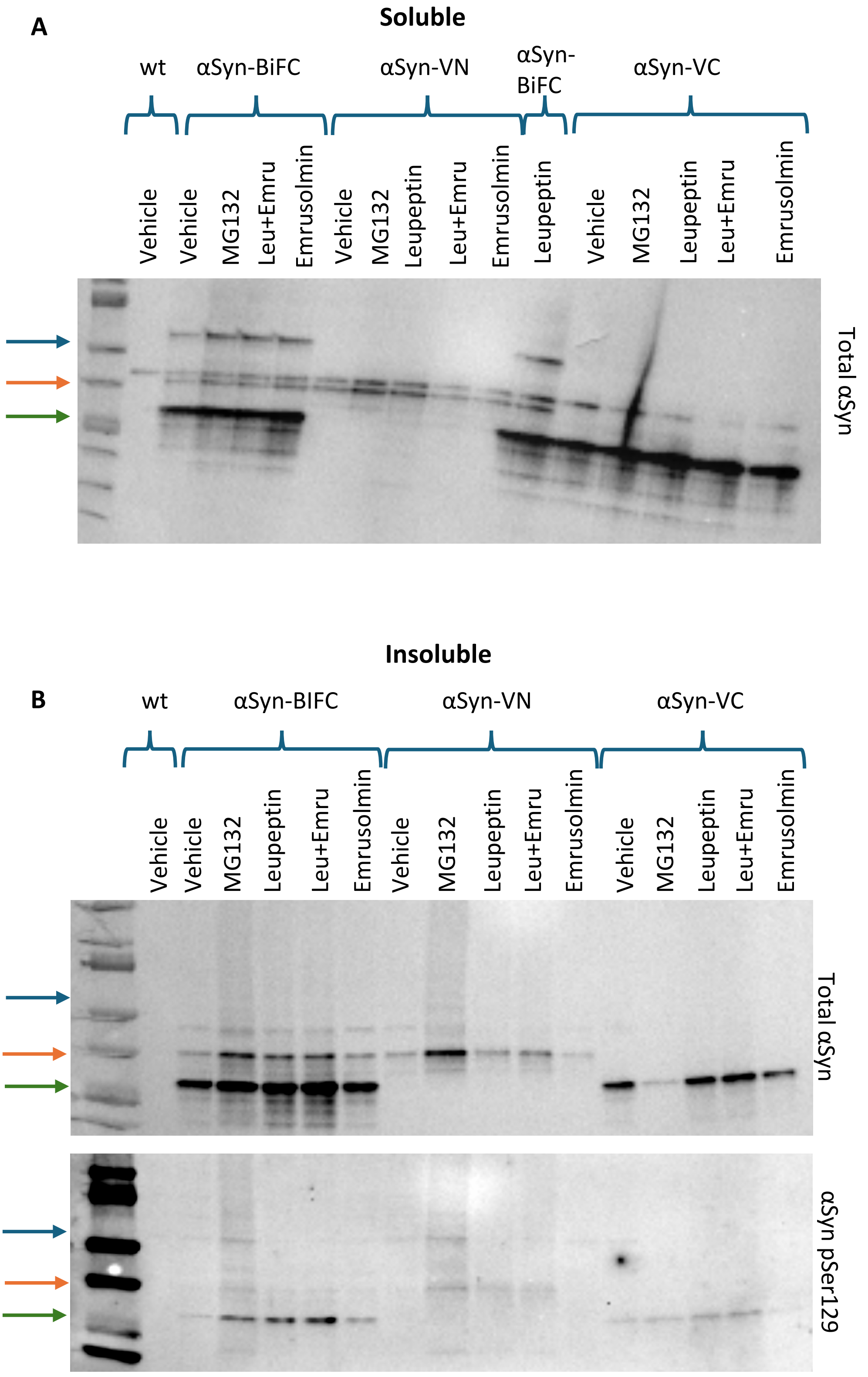

Supplementary Fig. 3. Aggregation-promoting treatments increase insoluble αSyn accumulation

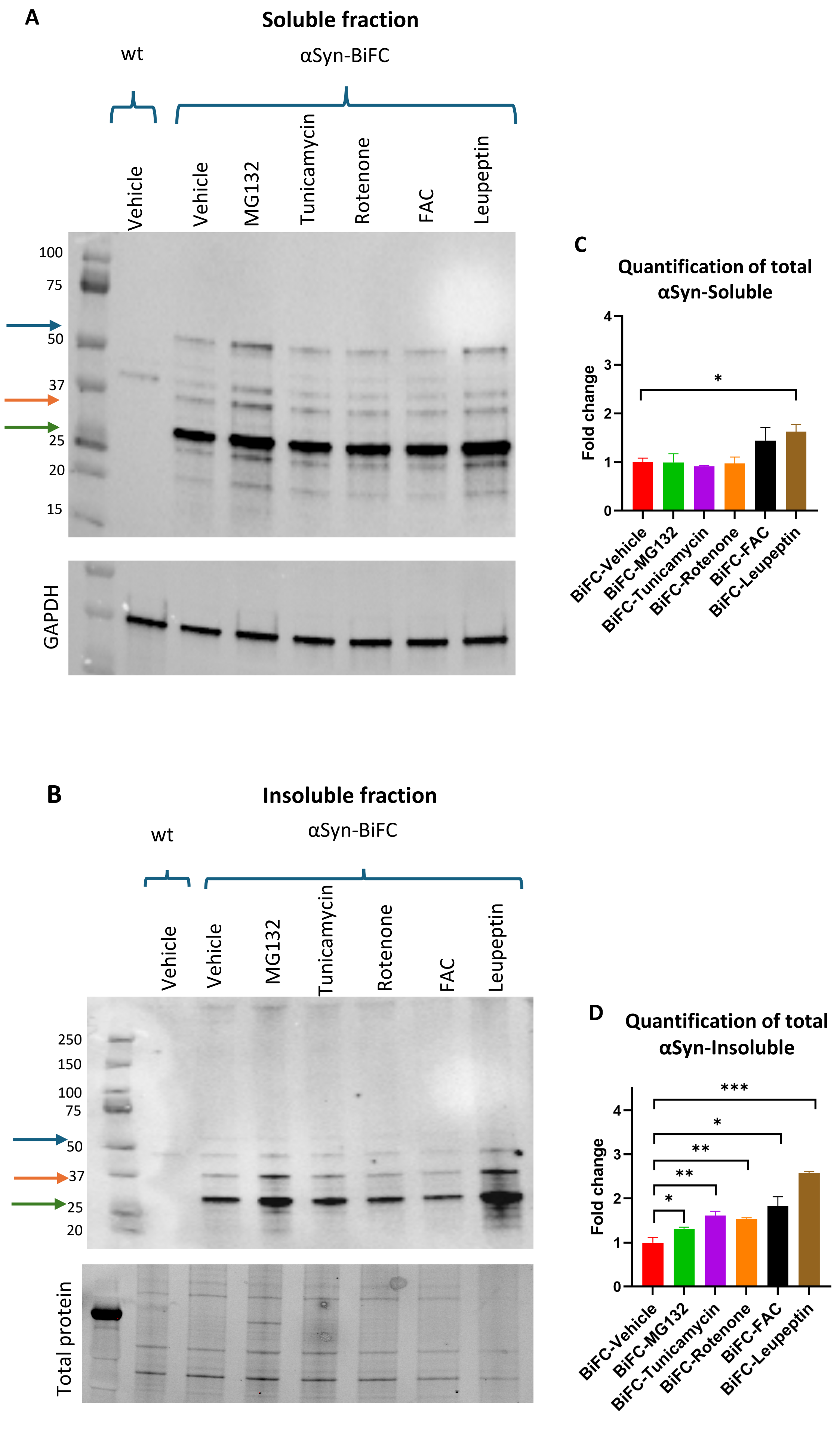

Supplementary Fig. 4: Emrusolmin reduces αSyn accumulation under Leupeptin challenge conditions

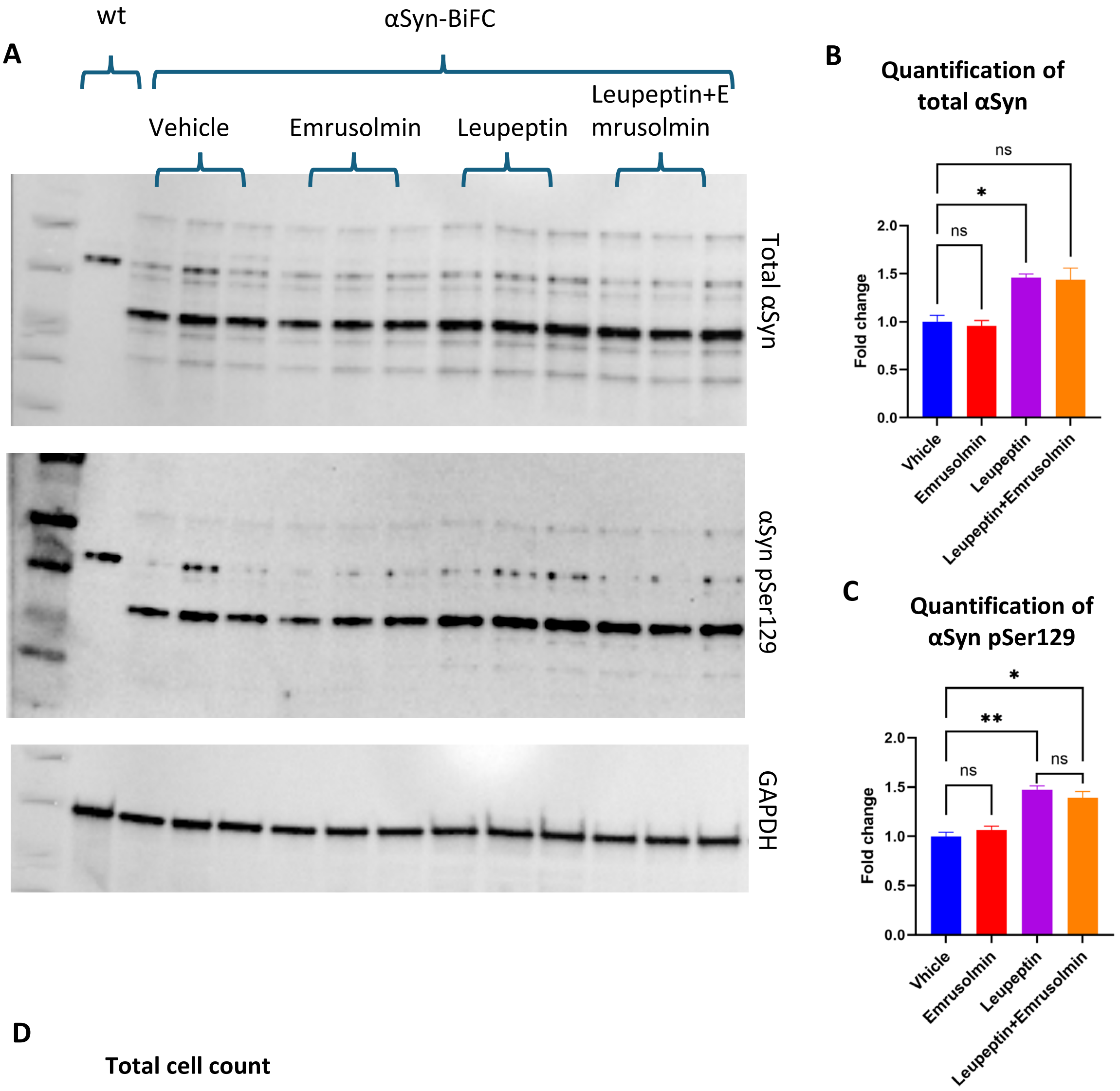

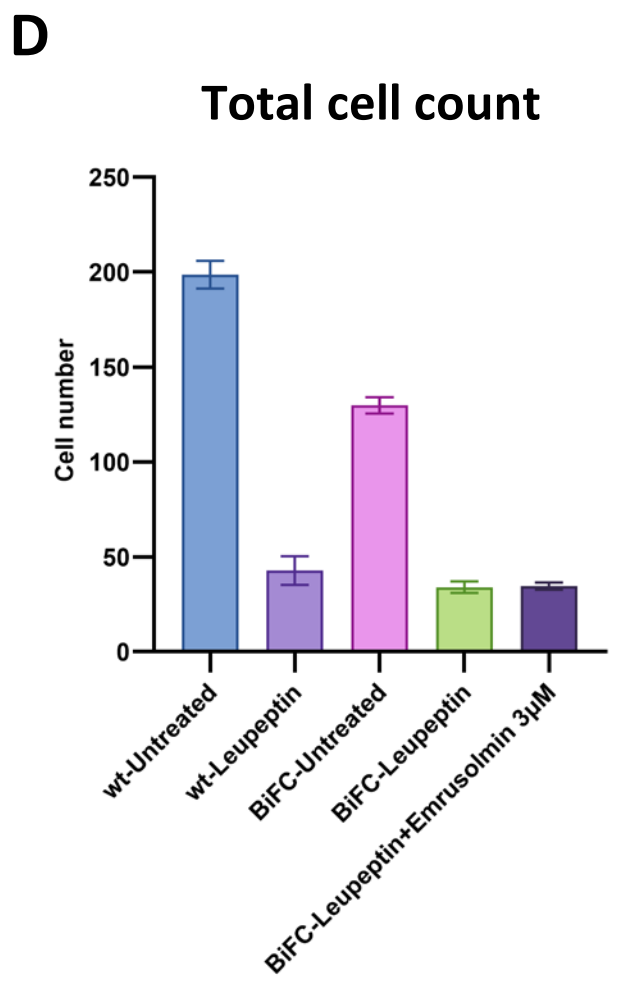

Supplementary Fig. 1: Robustness of αSyn-BiFC analysis across fluorescence intensity thresholds. Representative analysis of the same αSyn-BiFC dataset using four fluorescence-intensity thresholds spanning a broad range (500-1250 arbitrary units. a.u.). Real-time Venus fluorescence of untreated (green), MG132-treated (100 nM, purple; 500 nM, red) or leupeptin-treated (200μM, grey) U373-αSyn-BiFC cells over 72 hrs. Although the absolute percentage of Venus-positive cells varied according to threshold selection, the relative treatment-dependent effects remained consistent across all thresholds, demonstrating the robustness of the image-analysis approach.

Supplementary Fig. 2: Characterization of αSyn-BiFC, αSyn-VN and αSyn-VC cell lines and stability of fusion proteins. Wild-type (wt), αSyn-BiFC, αSyn-VN and αSyn-VC cells were treated with vehicle, MG132, Leupeptin, Emrusolmin, or Leupeptin + Emrusolmin and subjected to soluble/insoluble fractionation followed by Western blot analysis. **A** Representative Western blots of the soluble fraction probed for total αSyn. **B** Representative Western blots of the insoluble fraction probed for total αSyn and pSer129 αSyn. Blue arrows indicate the expected molecular weight of the αSyn-VN/αSyn-VC BiFC complex (~61 kDa), orange arrows indicate αSyn-VN (~35 kDa), and green arrows indicate αSyn-VC (~26 kDa).

Supplementary Fig. 3. Aggregation-promoting treatments increase insoluble αSyn accumulation. A Representative Western blots of soluble fractions and **B** insoluble fractions from αSyn-BiFC cells treated with vehicle, Leupeptin, MG132, Tunicamycin, Rotenone, or ferric ammonium citrate (FAC). Membranes were probed for total αSyn. GAPDH and total protein staining were used as loading controls for soluble and insoluble fractions, respectively. **C** Quantification of total αSyn levels in the soluble fraction (unpaired t-test with Welch's correction; Leupeptin vs. vehicle $p<0.05$; all other comparisons ns). Data represent mean ± SEM (n = 3 per group). **D** Quantification of total αSyn levels in the insoluble fraction (unpaired t-test with Welch's correction; MG132 vs. vehicle $p<0.05$, Tunicamycin vs. vehicle $p<0.01$, Rotenone vs. vehicle $p<0.01$, FAC vs. vehicle $p<0.05$, Leupeptin vs. vehicle $p<0.001$). Data represent mean ± SEM (n = 3 per group). Blue arrow: expected molecular weight of the αSyn-VN/αSyn-VC BiFC complex (~61kD). Orange arrow: expected molecular weight of αSyn-VN (~35kD). Green arrow: expected molecular weight of αSyn-VC (~26kD).

Supplementary Fig. 4: Emrusolmin reduces αSyn accumulation under Leupeptin challenge conditions. A Representative Western blot analysis of total αSyn in αSyn-BiFC cells treated with vehicle, Emrusolmin, Leupeptin, or Leupeptin + Emrusolmin. **B** Quantification of total αSyn levels (Welch's one-way ANOVA with Dunnett's correction; Vehicle vs. Leupeptin $p < 0.05$; all other comparisons vs. vehicle, ns). Data represent mean ± SEM (n=3). **C** Quantification of pSer129 αSyn levels (Welch's one-way ANOVA with Dunnett's correction; Vehicle vs. Emrusolmin ns; Vehicle vs. Leupeptin $p < 0.005$; Vehicle vs. Leupeptin + Emrusolmin $p < 0.05$; Leupeptin vs. Emrusolmin $p < 0.005$; Leupeptin vs. Leupeptin + Emrusolmin ns). Data represent mean ± SEM (n=3). **D** Quantification of total cell number at the 144 h endpoint of the therapeutic compound experiment shown in Fig. 6C, determined by Hoechst-stained nuclei counts following fixation. Although Leupeptin treatment reduced cell number relative to vehicle-treated cells, co-treatment with Emrusolmin did not further reduce cell number, indicating that the reduction in Venus-positive cells is unlikely to be explained by additional cell loss.
